# A systematic review on the application, phytochemistry and pharmacological activities of the genus *Atractylodes*

**DOI:** 10.1186/s13020-026-01473-2

**Published:** 2026-08-21

**Authors:** Siwei Luo, Jiameng Jiang, Dongmei Zhang, Ligen Lin, Qingwen Zhang

**Affiliations:** 1 State Key Laboratory of Mechanism and Quality of Chinese Medicine, University of Macau, Taipa, Macau SAR China; 2 Institute of Chinese Medical Sciences, University of Macau, Taipa, Macau SAR China; 3 State Key Laboratory of Bioactive Molecules and Druggability Assessment, Guangdong Basic Research Center of Excellence for Natural Bioactive Molecules and Discovery of Innovative Drugs, Jinan University, Guangzhou, 510632 China; 4 College of Pharmacy, Jinan University, Guangzhou, 510632 China

**Keywords:** *Atractylodes*, Application, Phytochemistry, Pharmacological activities

## Abstract

The genus *Atractylodes* (Asteraceae) is widely distributed and utilized in East and Southeast Asia, particularly in China, Japan, Korea and Thailand. In traditional Chinese medicine, representative species such as *A. lancea* and *A. macrocephala* have historically been employed to strengthen the spleen and eliminate dampness. Phytochemical investigations have led to the isolation and identification of 371 compounds from this genus, primarily encompassing polyacetylenes, terpenoids, flavonoids, phenylpropanoids, lignans, and their corresponding glycosides. In recent decades, extensive research has been conducted on the chemical constituents and pharmacological activities of plants within the genus *Atractylodes*. Concurrently, these crude extracts and isolated compounds have been demonstrated to exhibit diverse pharmacological activities, including anti-cancer, anti-inflammatory, gastrointestinal-regulatory, neuroprotective, hepatoprotective, and lung-protective activities. Regarding safety, their potential toxicity is closely tied to the traditional "dryness" property, which can be effectively mitigated through specialized processing methods such as bran-frying. However, rapidly accumulating data since 2021 has created a critical gap, rendering previous reviews insufficient to reflect the current research landscape. To address these emerging updates, this review systematically organizes recent advances in the traditional uses, phytochemistry, pharmacology, and safety profiles of the genus *Atractylodes*. Crucially, a critical analysis of structure–activity relationships is integrated to provide concrete, structured guidelines for future mechanistic exploration and rational drug development.

## Introduction

**Fig. 1:**
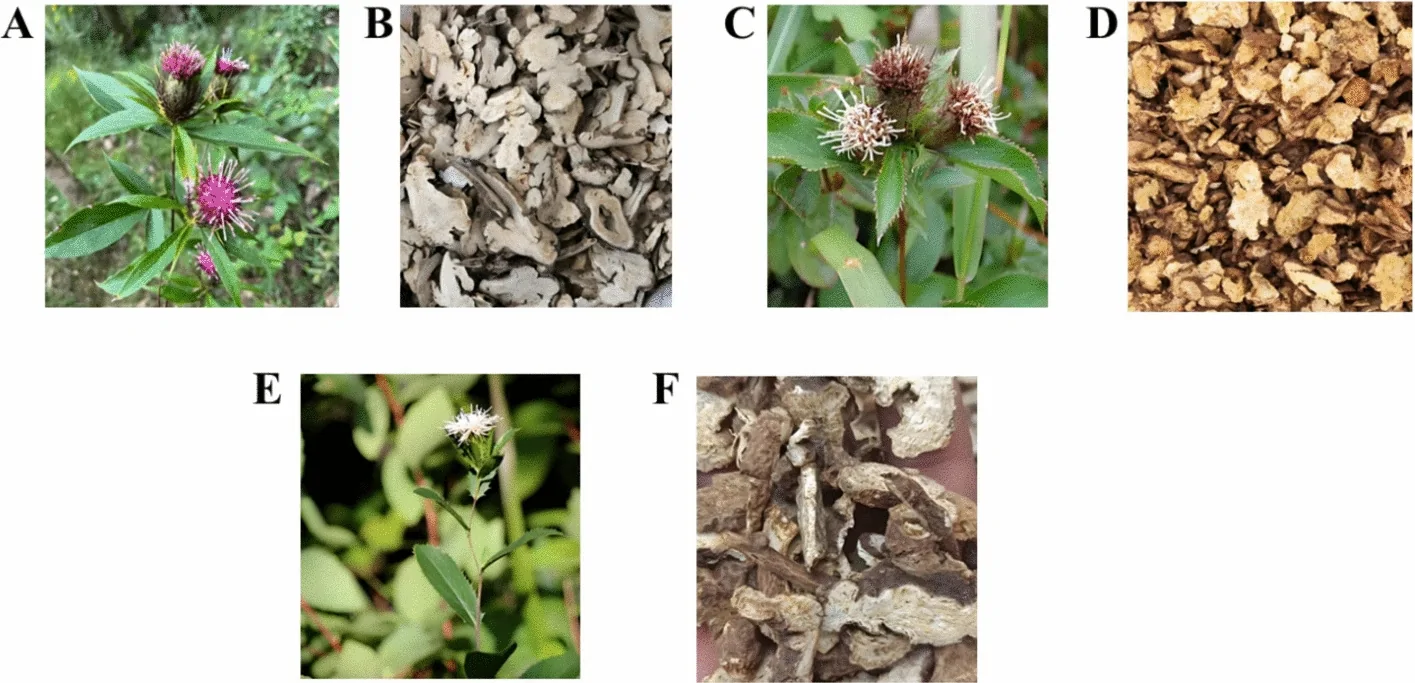
The whole plants of *A. macrocephala* (**A**), rhizomes of *A. macrocephala* (**B**), the whole plants of *A. lancea* (**C**), rhizomes of *A. lancea* (**D**), the whole plants of *A. chinensis* (**E**), rhizomes of *A. chinensis* (**F**)

*Atractylodes* is a prominent genus within the Asteraceae family, comprising perennial herbs that are widely distributed across East and Southeast Asia, primarily in China, Japan, and Korea. This genus includes seven species, among which *A. lancea*, *A. chinensis* and *A. macrocephala* are most notable [1]. In contrast, other species such as *A. japonica*, *A. ovata*, and *A. carlinoides* exhibit relatively limited geographic distributions, and consequently, their phytochemical and pharmacological profiles remain poorly understood Fig. 1. In TCM, the medicinal use of *A. lancea* was first documented in the Shennong's Herbal Classic, historically serving as a core therapy for night blindness, stomach pain, and influenza [2]. Similarly, the Japanese and Korean pharmacopoeias prescribe *A. lancea* as traditional diuretics and gastroprotective agents [3], while in Thailand, the plants of the genus *Atractylodes* are primarily used for the treatment of fever and colds [4].

From 1974 to 2025, systematic phytochemical investigations into the genus *Atractylodes* have led to the isolation and identification of 371 compounds, primarily characterized as sesquiterpenes and polyacetylenes. Additionally, lignans, flavonoids, and benzene derivatives constitute vital chemical components of this genus. With recent advancements in NMR spectroscopy, X-ray diffraction analysis, chemical synthesis and computational chemistry, the complex chemical structures of these metabolites have been unambiguously elucidated [5]. Concurrently, modern pharmacological studies have revealed that the crude extracts and isolated pure compounds from *Atractylodes* species possess promising bioactivities, including anti-cancer, anti-inflammatory, gastrointestinal function-improving, neuroprotective, hepatoprotective, and lung-protective effects [6–11]. Crucially, sesquiterpenes and polyacetylenes have been identified as the primary drivers of these diverse therapeutic actions.

Despite the extensive undocumented history and demonstrated therapeutic efficacy of *Atractylodes* species, several critical research bottlenecks persist. Although previous investigations have characterized the dominant volatile profiles and traditional processing variations of these herbs, the resulting quality control standards remain inconsistent, and systematic evaluations of the newly discovered complex compounds remain profoundly inadequate. Specifically, with the recent explosion of newly isolated chemical structures, the SAR governing their diverse pharmacological properties have not been fully elucidated, leaving the precise chemical basis for their multi-target effects partly ambiguous. Furthermore, while preclinical biological screenings of crude extracts have progressed rapidly, high-quality studies detailing the explicit molecular targets, in vivo metabolic forms, and robust clinical translation evidence of these pure constituents is still severely limited.

Although two systematic reviews addressing the phytochemistry and pharmacology of *A. macrocephala* and Atractylodis Rhizoma were published respectively by Zhu et al. and Zhang et al. [4, 12], numerous pioneering discoveries since 2021 have significantly expanded this field, rendering prior reviews insufficient to reflect the current research landscape. In recent years, novel meroterpenoids and sesquiterpene dimers with unique carbon skeletons have been characterized [13, 14]. Pharmacologically, extensive investigations have been conducted on the hepatoprotective and lung-protective effects of crude extracts and isolated compounds from this genus, particularly focusing on AT-I and AT-III [10, 15]. To address these emerging updates and unresolved challenges, this review provides a timely, comprehensive overview of the traditional uses, phytochemistry, and pharmacology of the genus *Atractylodes*. More importantly, a critical analysis of SAR and current research limitations is incorporated to establish structured guidelines for future research.

## Distributions and applications of* Atractylodes*

The genus *Atractylodes* represents a group of medicinal plants of significant importance in East Asia, primarily for their rhizomes. *A. carlinoides* is an exception because its rhizome is underdeveloped [16]. Currently, three species are recorded in the Chinese Pharmacopoeia. The historical record of these plants traces back to the Dong Han dynasty, where Shennong's Herbal Classic (《神农本草经》, A.D. 25–220) recorded "Zhu" as a single, top-grade medicinal entity without species differentiation [17]. A critical differentiation emerged during the Southern and Northern Dynasties, when Tao Hongjing’s Variorum of Classic of Herbology (《本草经集注》, A.D. 502–557) first systematically described two species based on botanical morphology. One was "Chizhu" (later known as "Cangzhu"), with slender leaves and bitter rhizomes better suitable for decoctions, the other was "Baizhu", characterized by large leaves and sweet rhizomes more appropriate for pills and powders [12]. And the first systematic differentiation of their efficacy and clinical applications was recorded in Amplified Herbology (《本草衍义》) (Song Dynasty, A.D. 960–1127), a differentiation that has been refined and formalized over subsequent centuries [18].

According to the 2020 edition of the *Chinese Pharmacopoeia*, Atractylodis Rhizoma ("Cangzhu") derives from the dried rhizomes of *A. lancea* and *A. chinensis*, while Atractylodis Macrocephalae Rhizoma ("Baizhu") originates solely from *A. macrocephala*. The geographical distributions of these source plants are notably distinct. *A. lancea* is primarily distributed in Jiangsu, Hubei, Henan, Anhui, and Zhejiang provinces, with rhizomes from the Maoshan region of Jiangsu being especially prized for their superior quality and recognized as the traditional geo-authentic source of "Cangzhu". *A. chinensis* is mainly produced in Hebei, Shaanxi, and Shanxi provinces, with the produce from Chengde, Hebei generally regarded as higher grade. *A. macrocephala* cultivation is concentrated in Zhejiang, Anhui, and Hebei provinces, among which the Yuqian region in Zhejiang being renowned for producing the highest grade [18, 19]. Regarding the minor species, their geographical distributions exhibit distinct regional characteristics. The wild resources of *A. japonica* (historically known as *A. ovata* in Japan) are widely distributed across Northeast China, Japan, and the Korean Peninsula. It is a traditional medicine for the Korean ethnic group and is used as "Cangzhu" in Japan and South Korea. *A. carlinoides* is strictly endemic to western Hubei province, China [20, 21].

**Fig. 2:**
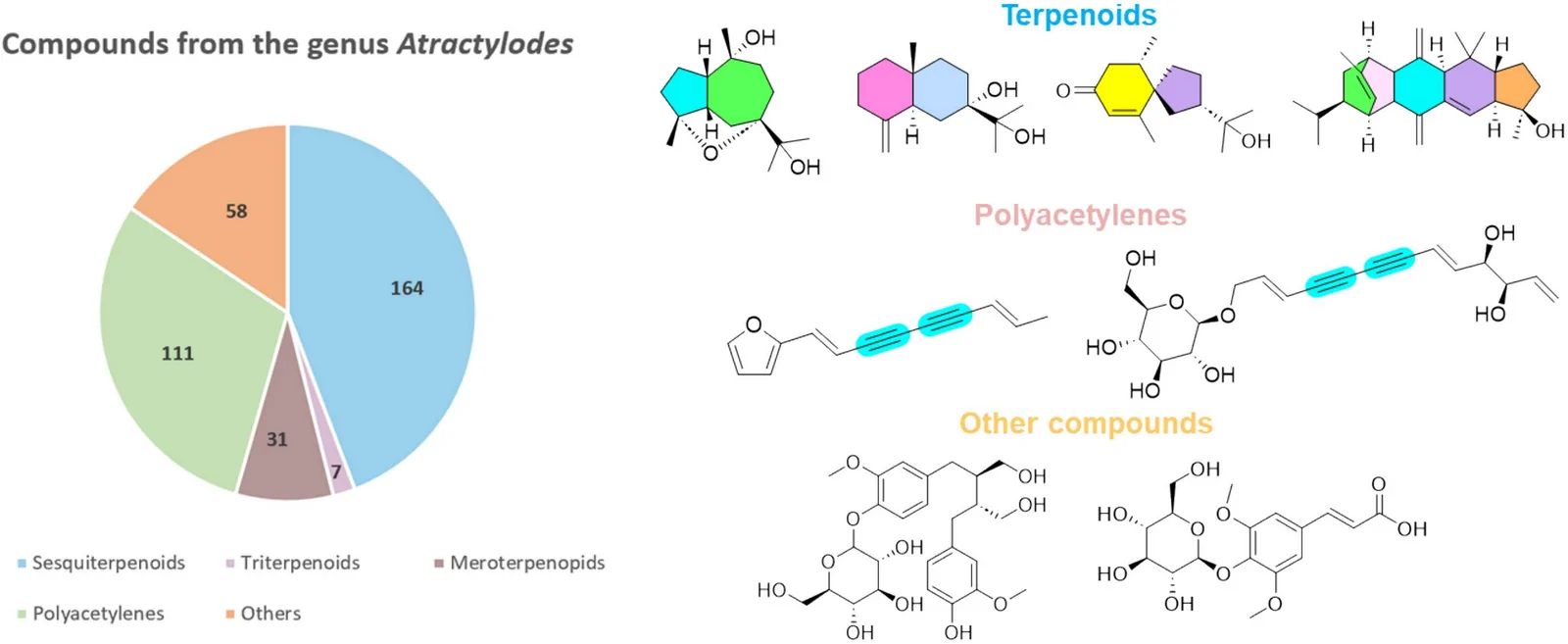
Types of the compounds from the genus *Atractylodes*

**Fig. 3:**
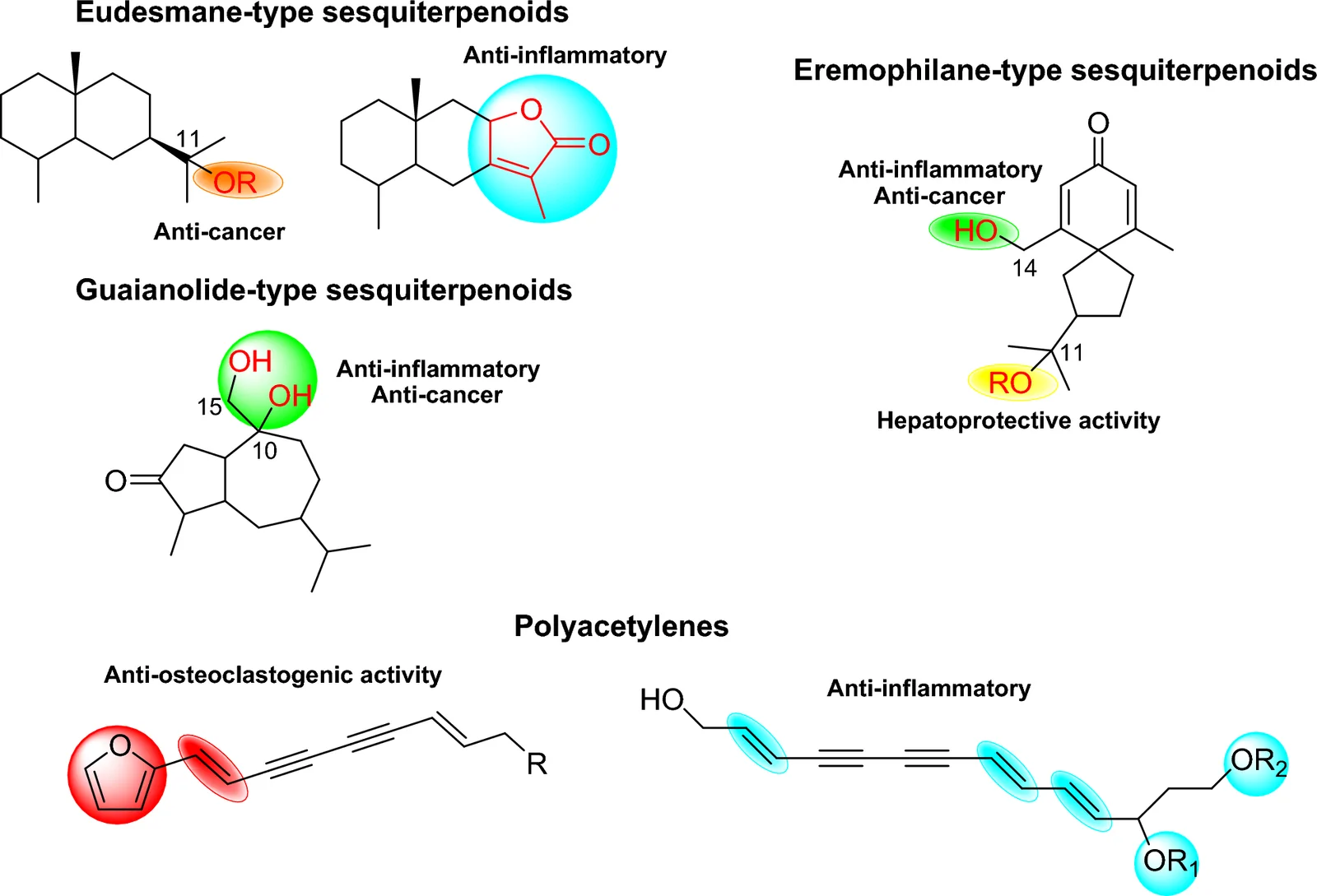
The SAR of sesquiterpenoids and polyacetylenes from the genus *Atractylodes*

**Fig. 4:**
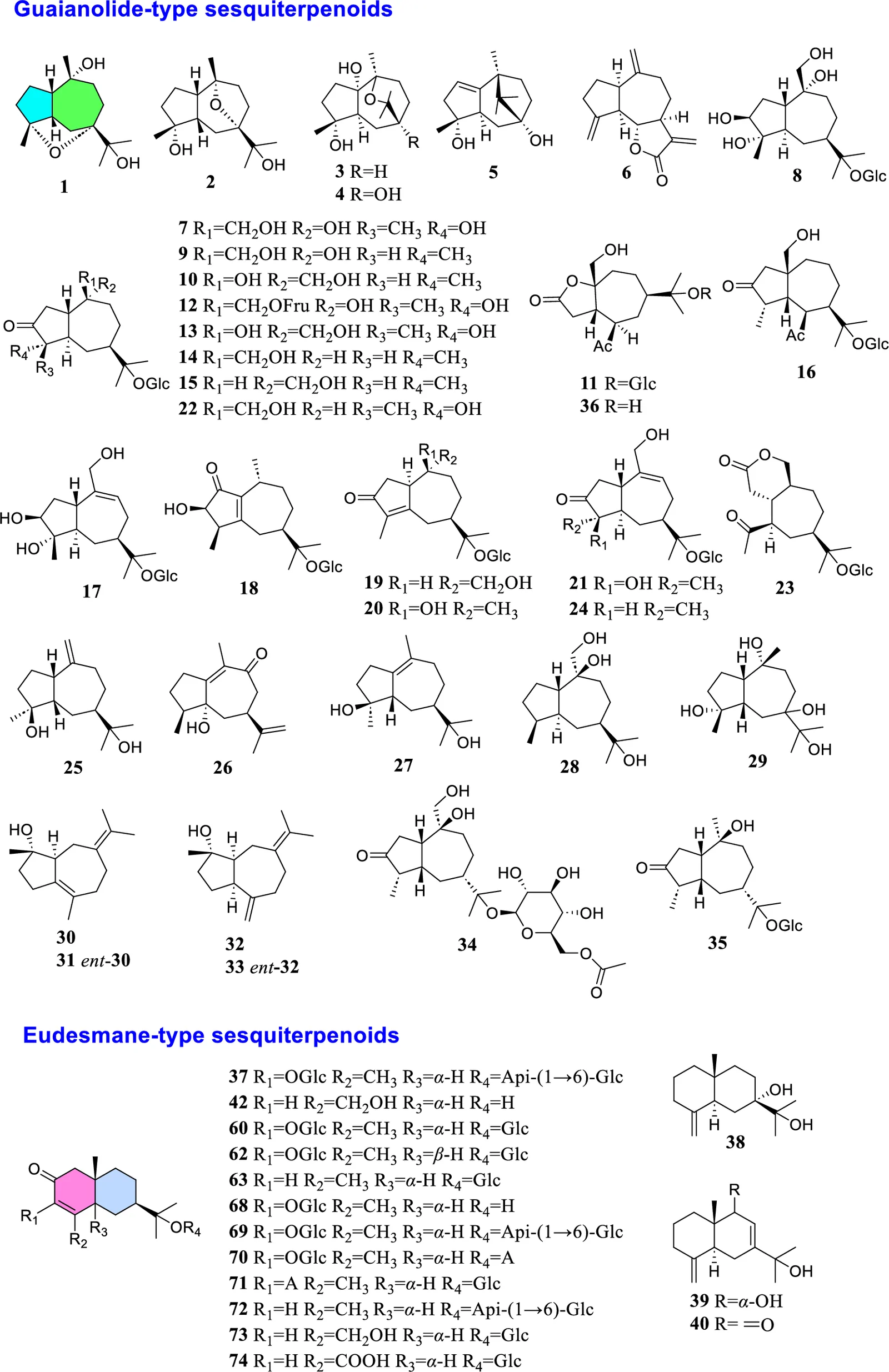

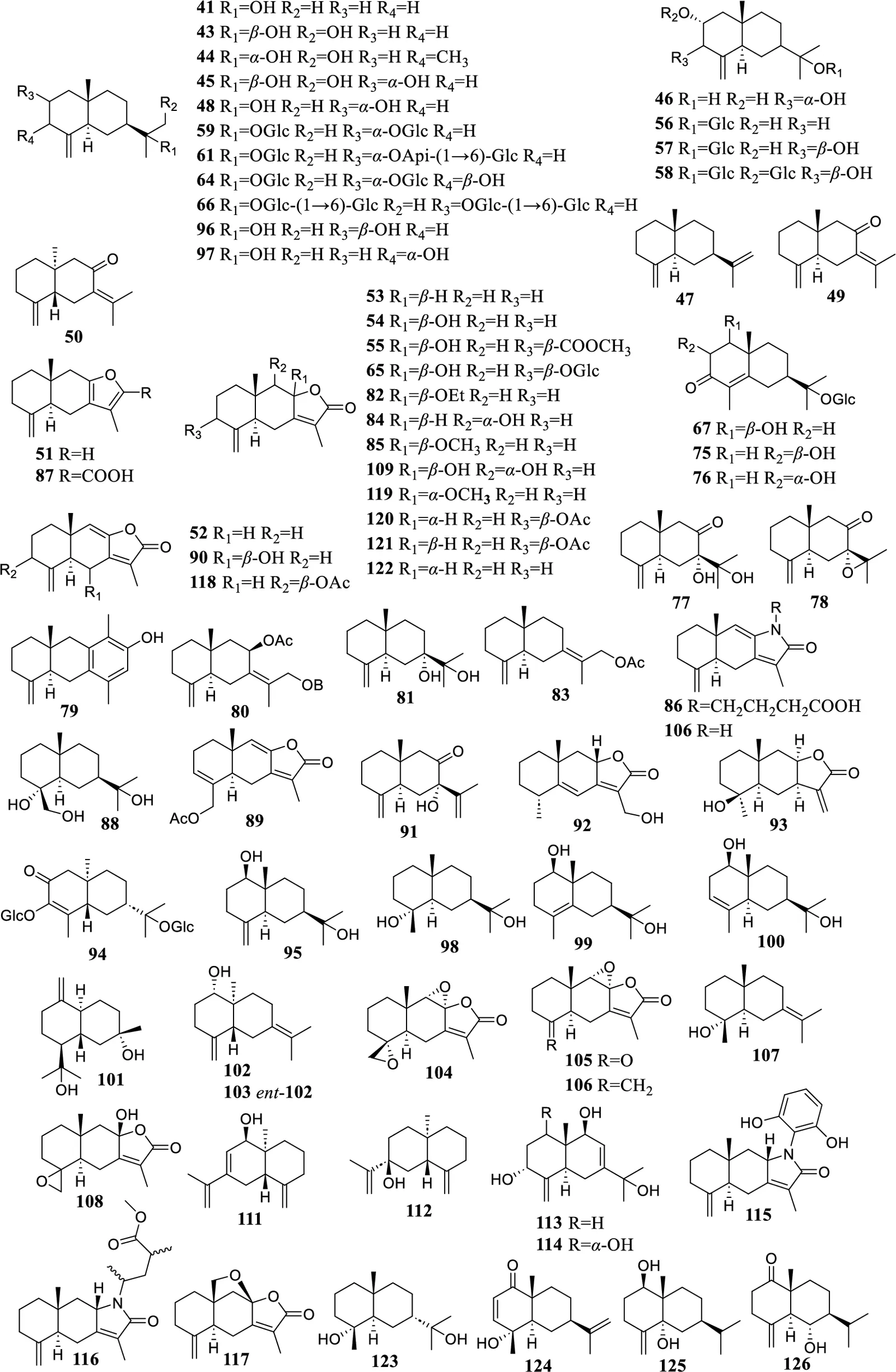

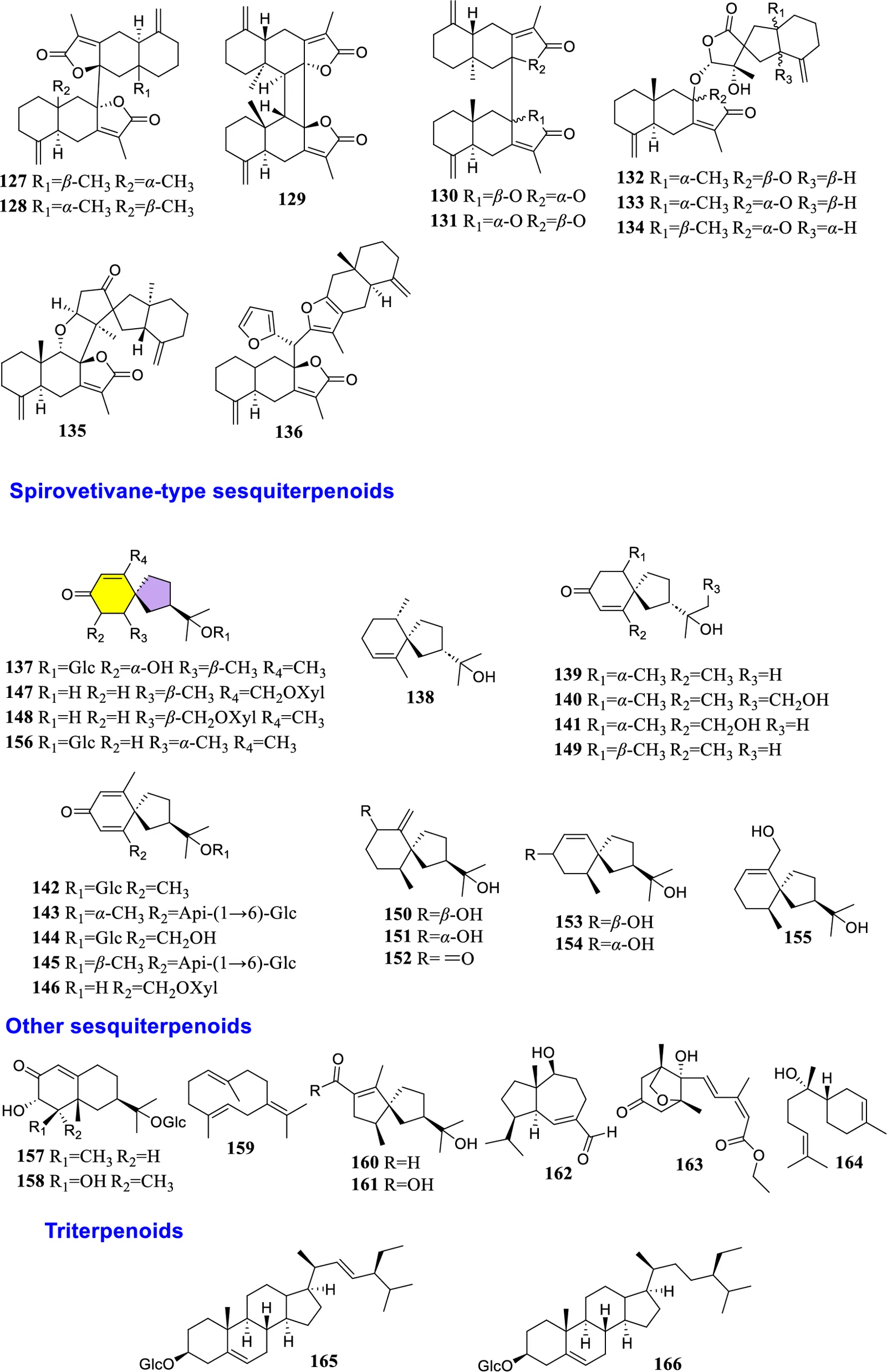

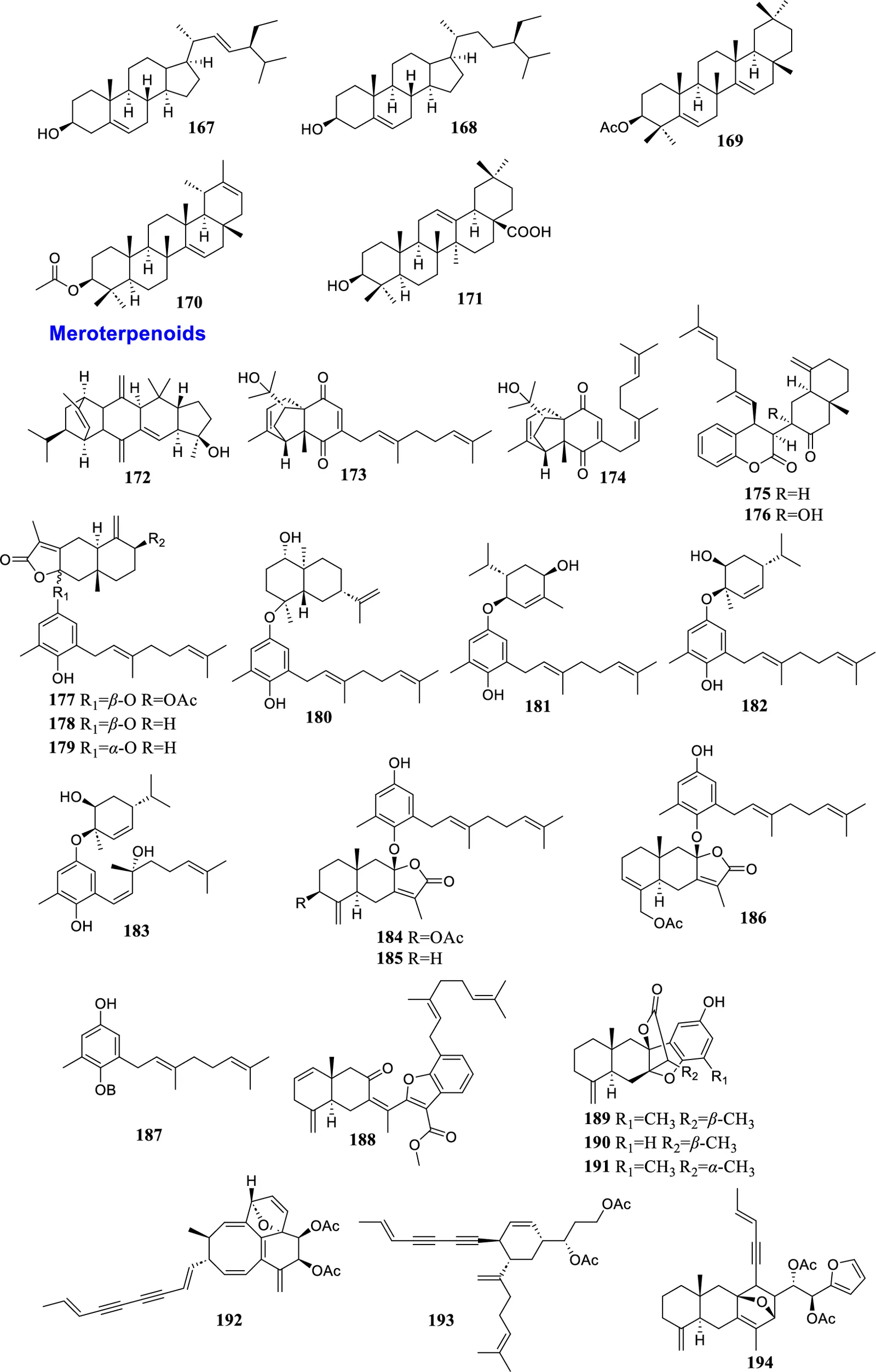

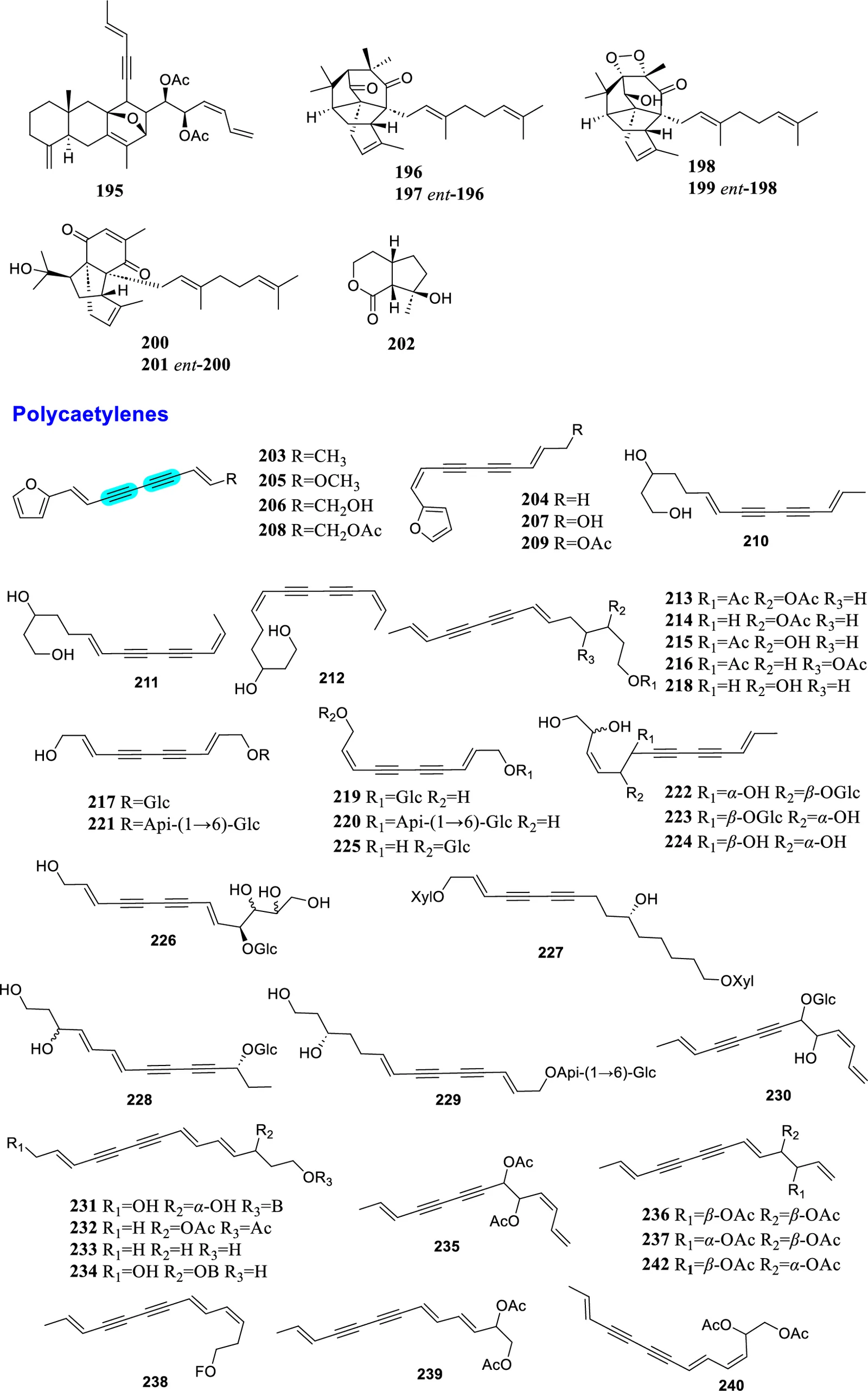

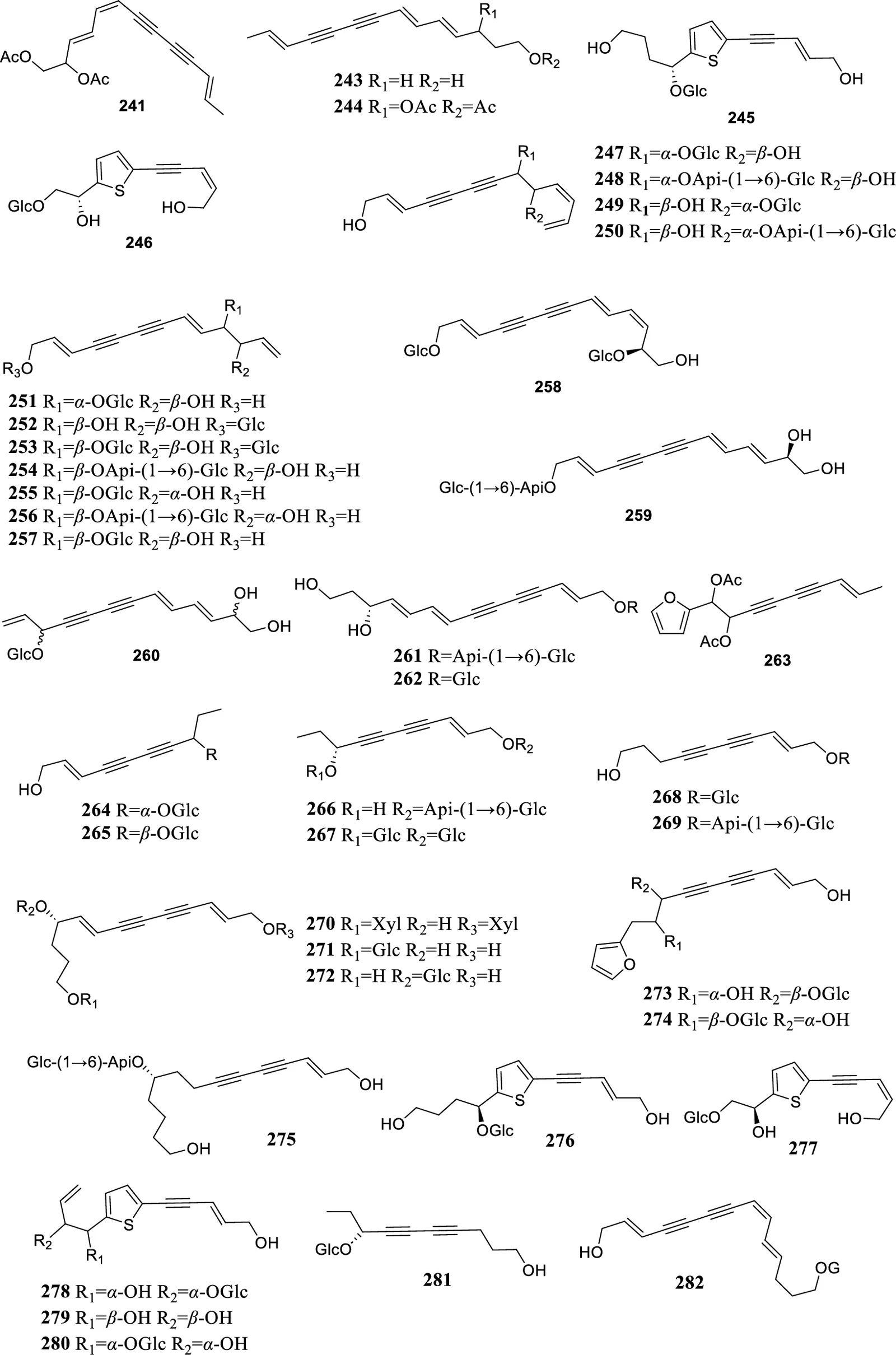

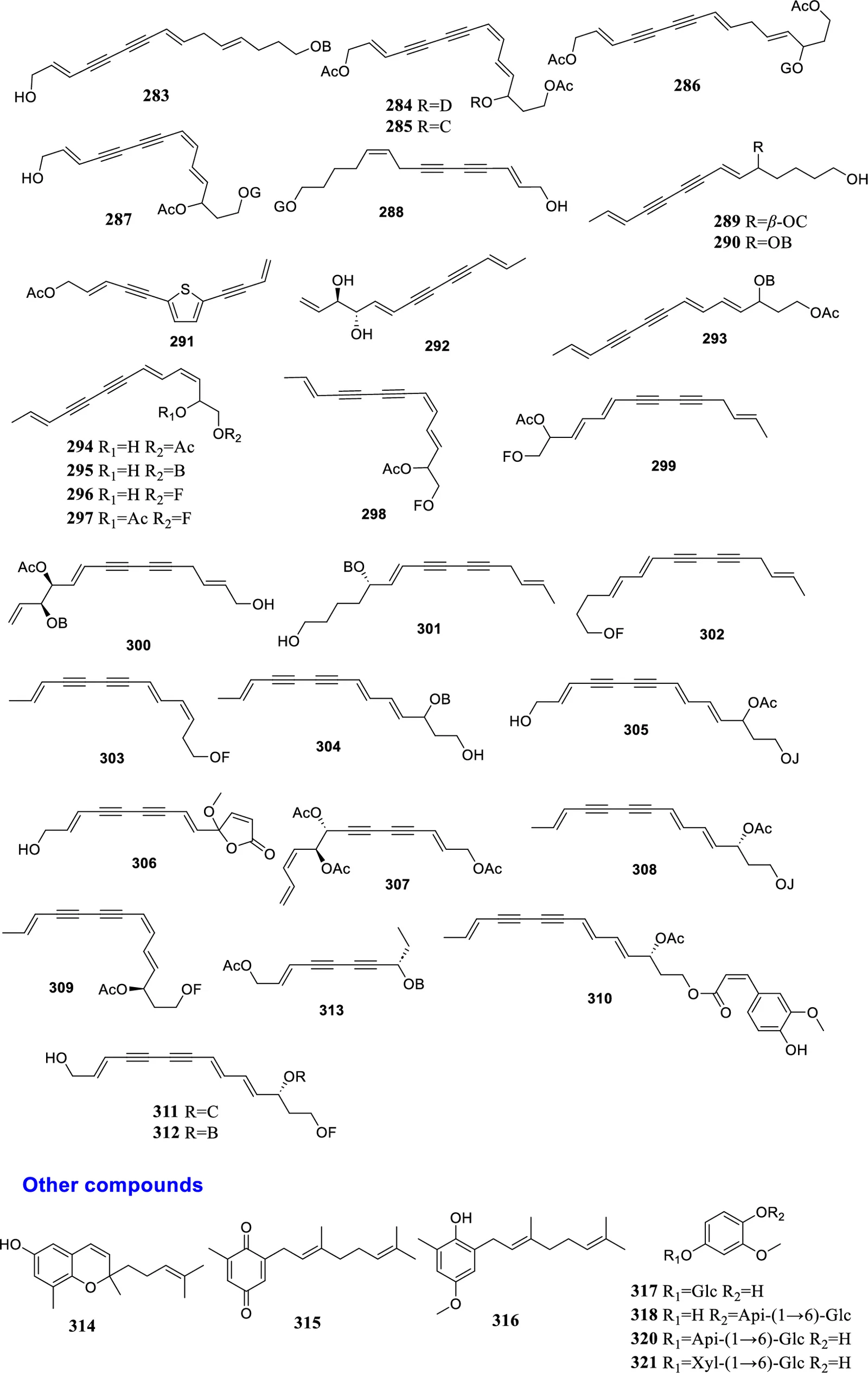

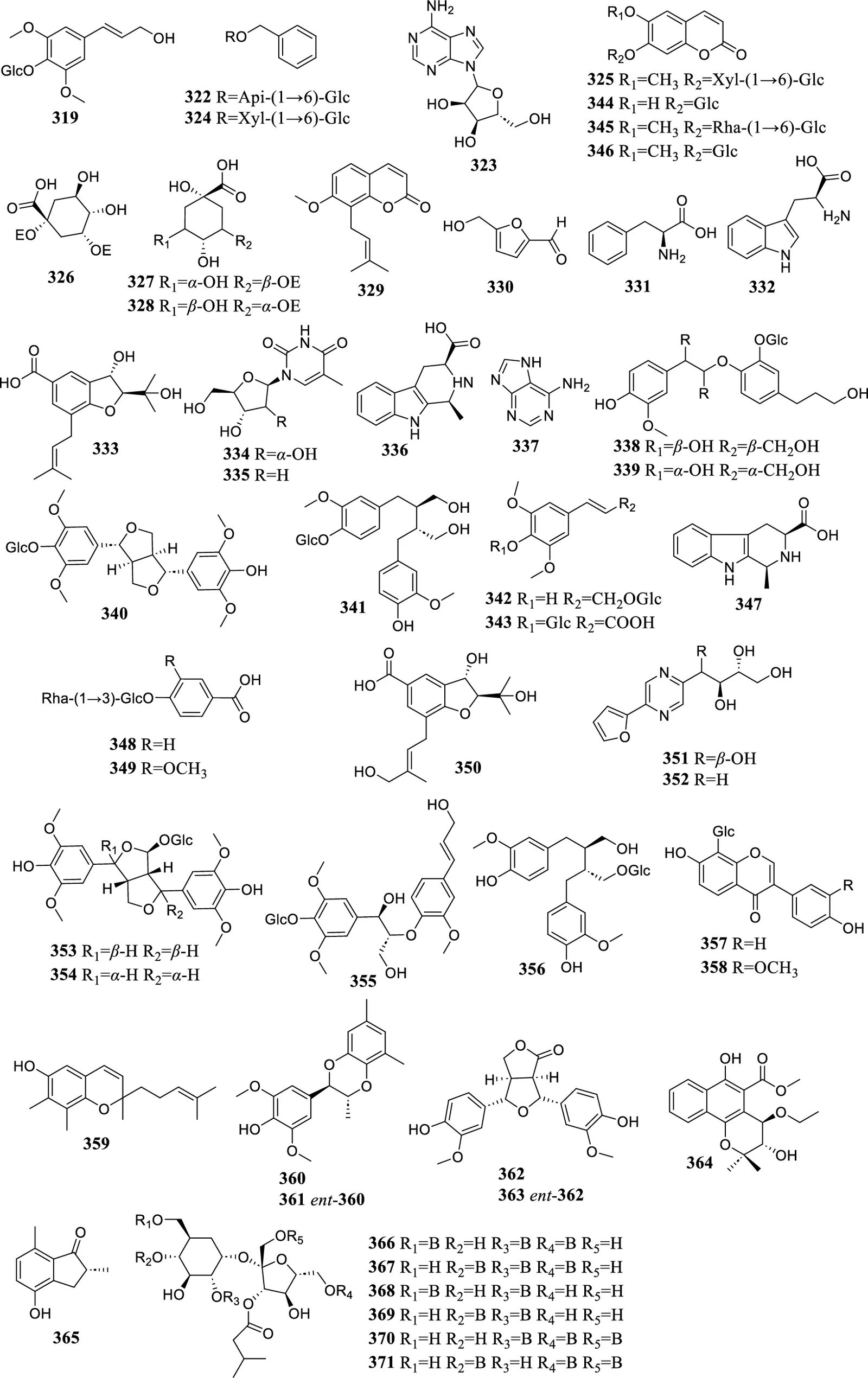

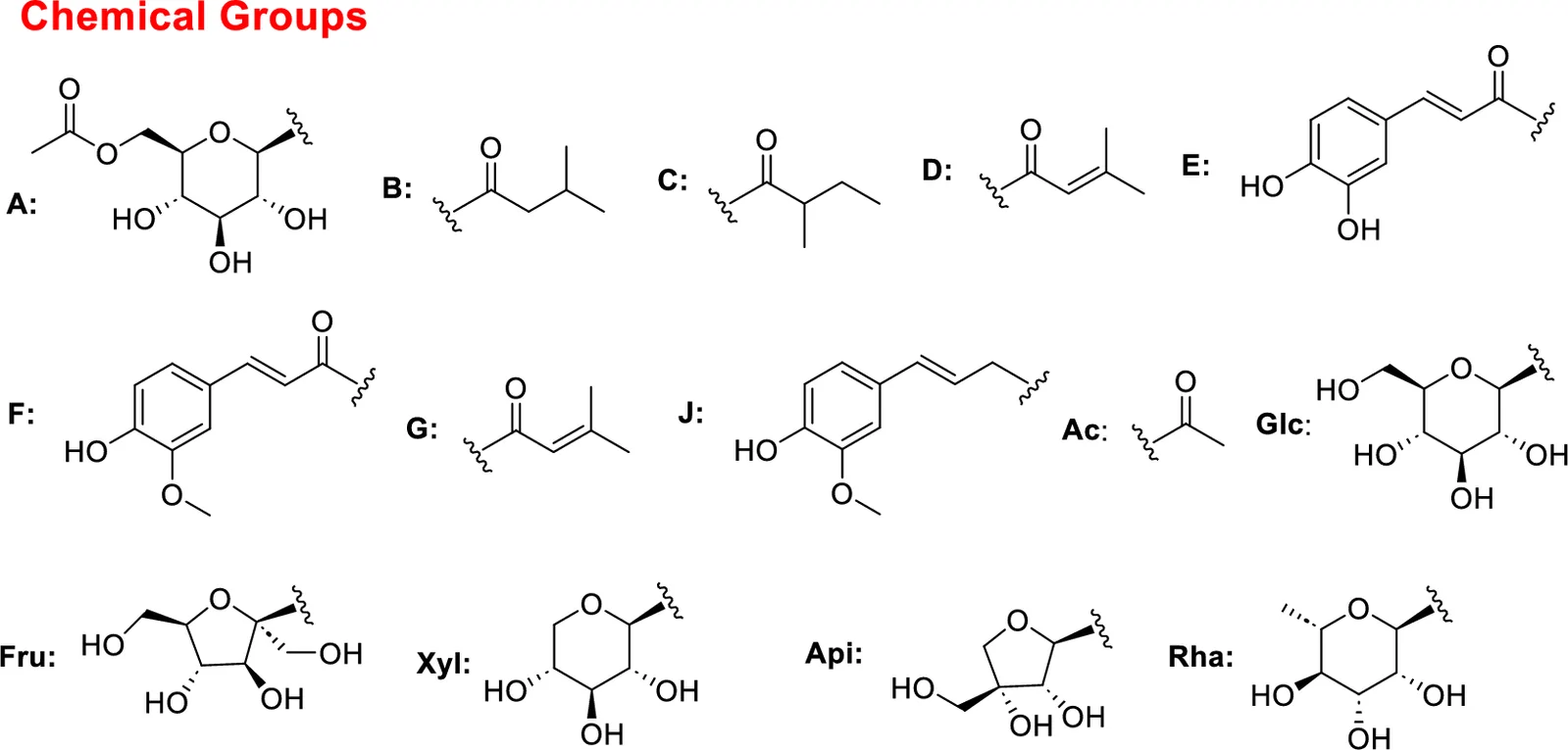
Structures of the compounds isolated from the genus *Atractylodes*

**Fig. 5:**
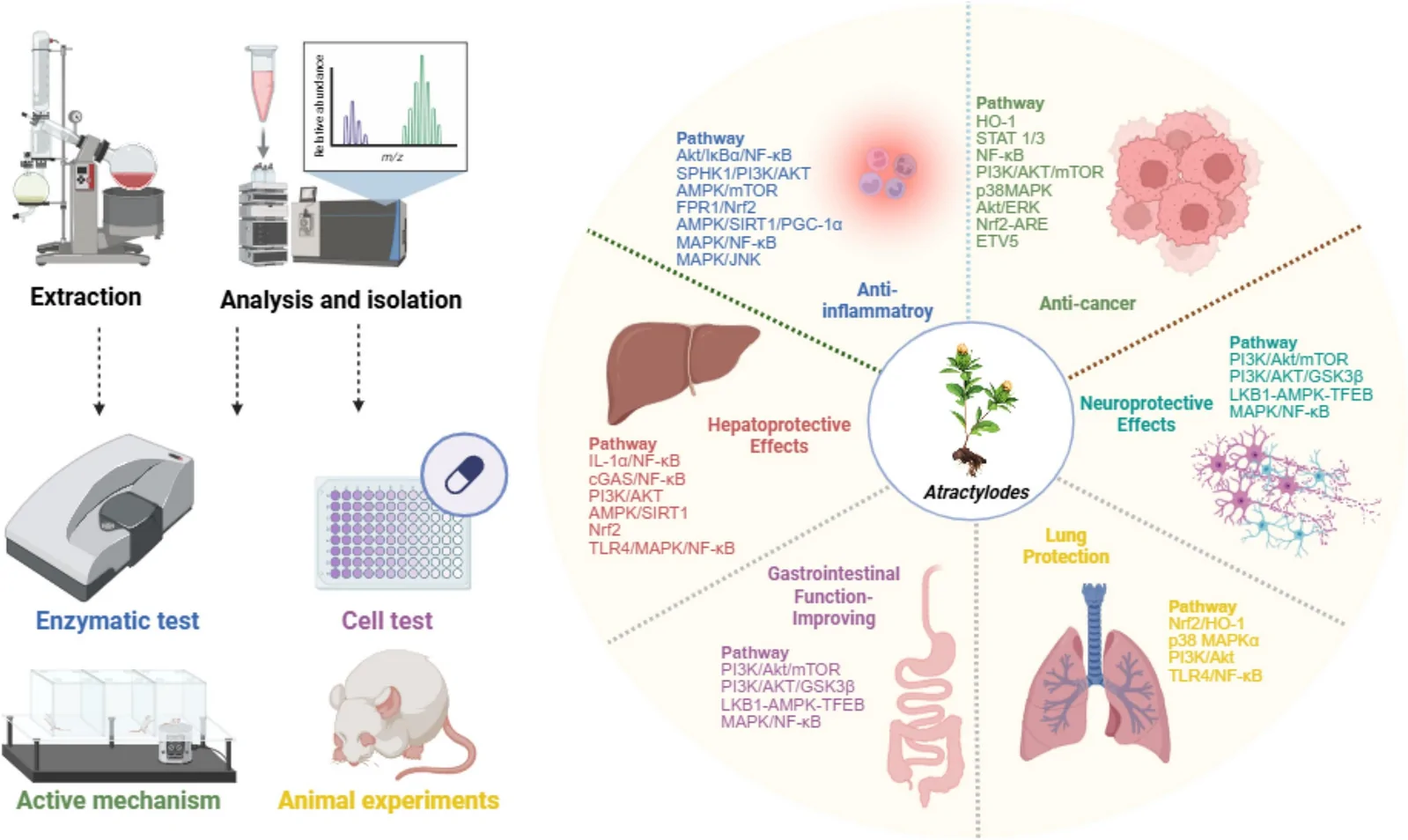
Overview of pharmacological effects from the genus *Atractylodes*

The pharmacopoeia also delineates their core therapeutic properties. Both "Cangzhu" and "Baizhu" are recognized for strengthening the spleen and eliminating dampness. However, "Cangzhu" is additionally noted for improving vision, "Baizhu" is emphasized for arresting sweating and calming the fetus [22]. Commonly, *Atractylodes* rhizomes should be processed to modulate their medicinal properties and therapeutic efficacy. For *A. macrocephala*, the 2020 edition of the Chinese Pharmacopoeia records bran-fried *A. macrocephala*, a process that facilitates the chemical transformation of atractylon into atractylenolides while decreasing volatile oils and mitigating the inherent dryness, thereby enhancing its gastrointestinal-regulatory and anti-inflammatory activities [23, 24]. In contrast, earth-fried *A. macrocephala* exhibits an enrichment of polysaccharides and lactones, which potentially reinforces its immunomodulatory and neuroprotective effects [25, 26]. For *A. lancea*, stir-frying to yellow aims to diminish dryness, stir-frying to scorched enhances intestinal barrier protection and antidiarrhea, and stir-frying to charcoal shifts the property toward hemostasis [27]. Additionally, processing with rice-water or bran both attenuates dryness while respectively reinforcing spleen-strengthening or gastric-toning effects, earth-frying optimizes spleen-strengthening antidiarrhea, whereas salting and wine-processing steer the efficacy toward diuresis and blood circulation, respectively [27]. Mechanistically, *A. lancea* processing typically downregulates volatile components such as *β*-eudesmol and atractylodin to relieve gastric irritation and dryness [28], while simultaneously upregulating non-volatile constituents including *β*-daucosterol, atractyloside A, and polysaccharides, which directly correspond to its anti-inflammatory, spleen-strengthening, anti-diarrheal, immunomodulatory, and intestinal barrier protective activities [29]. In general, processing is not just about "reducing toxicity" or "increasing efficacy” but rather reconfigures the medicine's properties and therapeutic focus according to clinical needs (Figs. 1, 2, 3, 4, 5).

Beyond China, *Atractylodes* species are widely integrated into other Asian traditional medical systems. In Japan, the dried rhizome of *A. lancea*, termed "Sojutsu", is a fundamental kampo medicine constituent (e.g., Juzen-taiho-to and Saireito) utilized as a diuretic and gastric toner [30–32]. Additionally, the rhizome of *A. macrocephala* has been historically employed in Japanese traditional medicine to treat ulcer‑related conditions, including gastric mucosal hemorrhage [33]. In Thailand, *A. lancea* (locally known as "Khod-Kha-Mao") is predominantly applied to treat fever, common colds, and non-infectious gastrointestinal distress [34, 35]. In Korea, *A. macrocephala* ("Baek-chu") is traditionally prescribed to alleviate abdominal pain, edema, and obesity, and serves as a key ingredient in formulas like Soamsan to aid chronic disease remission [36, 37].

## Phytochemistry

The plants of the genus *Atractylodes*, which are rich in diverse chemical components, mainly including terpenoids and polyacetylenes. From 1974 to 2025, researchers have isolated 371 compounds from the genus *Atractylodes*, including 202 terpenoids and 111 polyacetylenes Fig 2, 4 Table 1. Pharmacological studies have demonstrated that these compounds represent important active constituents of the genus *Atractylodes*. Notably, sesquiterpenoid glycosides and polyacetylene glycosides account for nearly half of all the reported compounds from this genus, highlighting the significant value of polar compounds in *Atractylodes* plants for phytochemical research. This review systematically summarizes the structures of compounds isolated from plants of the genus *Atractylodes*, providing a reference and guidance for the research in future.

### Sesquiterpenoids

Sesquiterpenoids represent one of the most abundant chemical components which possess diverse structures due to their complex three-dimensional structures. To date, a total of 164 sesquiterpenoids have been reported, mainly including guaianolide-type, eudesmane-type and spirovetivane-type. Guaianolide-type sesquiterpenoids feature a 5/7 bicyclic framework, eudesmane-type possess a 6/6 bicyclic skeleton, and spirovetivane-type derivatives are characterized by a spiro [4, 5] ring system. In 2008, Wang et al. isolated and purified a series of the guaianolide-type sesquiterpenoids containing an epoxy unit together with a tricyclic carbon skeleton sesquiterpenoid formed by guaianolide-type, their cytotoxic effects against P388 and A549 cell lines were subsequently evaluated [38]. Xu et al. conducted investigations on the *n*-Butanol fraction of *A. lancea* and identified twenty-five sesquiterpenoid glycosides of different skeleton types (**18–20**, **67–76**, **137**, **140–148**, **157**, **158**). In addition, pharmacological experiments in vitro revealed that several of them exhibited anti-inflammatory and liver-protective activities [1, 5, 39–41]. A total of 20 sesquiterpenoids (**25–27**, **95**–**101**, **138**, **139**, **150**–**155, 160, 161**) have been isolated from *A. lancea*, among them, two novel ones (**160**, **161**) possessed an unprecedented spiro [4, 4] skeleton system and their planar structures, relative configurations and absolute configurations were elucidated [42]. The bioassay-guided isolation was adopted by Zhou et al. and successfully identified **89**–**93** which showed obvious anti-inflammatory activity [43]. To date, only ten Sesquiterpene dimers have been reported which have been proven to exhibit significant anti-inflammatory and other pharmacological properties [13, 44, 45].

### Triterpenoids

Up to now, 7 triterpenoids (**165**–**171**) have been isolated from the genus *Atractylodes*, all of them were published by Duan et al. in 2008 for the first time [46]. Among them, **165** exhibited significant anti-cancer activities in HCT-116 and MKN-45 cell lines.

### Meroterpenoids

In 2021, Chen et al. reported three pairs of meroterpenoids (±)-atrachinenins A—C (**196**–**201**) with novel 6/6/5/5 tetracyclic system and 6/6/5/5/5 cyclic ring system, their biosynthesis pathways were proposed. Subsequently in 2022, The structures of atrachinenynes A–D (**192**–**195**) were charactered, **192** features an unusual 8/6/6/5 tetracyclic ring skeleton. Recently, sixteen new meroterpenoids atrachinenin D-S (**172**–**187**) were isolated and identified by them, **172**–**177** possesed an unprecendented tricyclic fragment skeletons, **177**–**187** have new oxygen-bridged hybrids [14, 47, 48]. Wen et al. reported a novel sesquiterpenoid-geranyl benzofuran conjugate (**188**) and evaluated the cytotoxicity in vitro [49]. In 2023, Zhang et al. isolated atramacronoids A-C (**189**–**191**) featuring an unusual 6/6/5/5/6 skeleton formed by the C-8-C-16 linkage [50].

### Polyacetylenes

As an important class of bioactive compounds, polyacetylenes significantly contribute to the pharmacological effects and chemical diversity of the genus *Atractylodes* plants. In addition to sesquiterpenoids, Xu’s research group also conducted investigations on polyacetylenes and found 47 polyacetylene glycosides (**219–229**, **245–262**, **264–281**) from *A. lancea*. More importantly, they proposed a method for quickly determining the configuration of hydroxyl groups in polyacetylene [1, 5, 40, 41, 51–53]. Sun et al. and Zhou et al. also discovered a series of polyacetylenes (**296**–**307**), and their chemical structures and pharmacological activities were further studied [42, 43]. Yao et al. isolated six novel polyacetylenes (**282**–**287**) from *A. macrocephala* and test the inhibitory activity against NO production in lipopolysaccharide-induced RAW264.7 cells [54].

### Other compounds

In addition to terpenoids and polyacetylenes, many other types of the compounds such as phenolic derivatives, lignans, flavonoids and alkaloids in the plants of the genus *Atractylodes*. In 2003, Kitajima et al. reported that *A. lancea* contained 7 aromatic compounds together with 2 amino acids (**317**–**323**, **331**, **332**) [55]. Xu et al. purified and identified 19 lignans, flavonoids and aromatic compounds (**324**, **325**, **342**–**358**) and identified their structures by NMR and ECD calculation [39, 41, 56]. Tanaka et al. investigated the chemical constituents of *A. lancea and* discovered six unprecedented acylsucrose derivatives (**366**–**371**) [57]. In 2023, Zhang et al. isolated and purified two pairs of lignans and two aromatic compounds (**360–365**). Among them, **360** and **361** are a pair of rare enantiomeric benzodioxane norneolignans, their absolute structures were determined by ECD calculation [58].

### The SAR of major constituents

In the genus *Atractylodes*, sesquiterpenoids and polyacetylenes serve as the main active ingredients, exhibiting a certain correlation between their structural characteristics and pharmacological effects Fig 3. Within the eudesmane-type sesquiterpenoids, glucosylation of the C-11 hydroxyl group leads to potentiation of the inhibitory activity against HepG2 cells, while the incorporation of an unsaturated five-membered lactone ring at the same position serves to augment the anti-inflammatory properties [39, 43]. As for the eremophilane-type sesquiterpenoids, the un-substituted C-14 position (maintaining the original methyl group) contributes to better anticancer activity, whereas hydroxylation at this site (converting to a hydroxymethyl group) favors the exertion of anti-inflammatory effects; meanwhile, their hepatoprotective activity is closely related to the glycosylation of the C-11 side chain, with the 11-OH substituted by Glc alone showing no hepatoprotection, whereas substitution by an Api-Glc disaccharide exhibits significant liver-protective effects [40, 59]. Regarding guaianolide-type sesquiterpenoids, simultaneous substitutions at both the C-10 and C-15 positions lead to an effective potentiation of their anti-inflammatory activity along with enhanced cytotoxicity against A549 cancer cells [60]. On the other hand, in the case of polyacetylenic compounds, the C_10_-polyacetylene constituents possessing a terminal furan ring and an *E*-configured double bond at the 8,9-position exhibit anti-osteoclastogenic activity; conversely, for C_14_-polyacetylene components, when the conjugated double bonds are all *E*-configured and both the R_1_ and R_2_ positions are substituted, their anti-inflammatory activity is significantly enhanced [54, 61].

## Pharmacology

Plants of the genus *Atractylodes* have a long history of medicinal use. Modern pharmacological investigations have demonstrated that extracts and compounds from this genus exhibit a variety of bioactivities, including anti-cancer, anti-inflammatory, neuroprotective, and gastrointestinal function-improving effects Fig 5.

### Anti-cancer effects

Natural products isolated from TCM are increasingly recognized for their clinical potential as anticancer lead compounds and complementary therapeutic strategies [87]. They have been shown to enhance tumor suppression and reverse or delay the development of tumor cell resistance to conventional chemotherapeutic agents. Extensive studies have demonstrated that extracts and active constituents from *Atractylodes* species exhibit multi-target anticancer effects in a variety of malignancies, including CCA, gastric cancer, lung cancer, liver cancer and breast cancer Table 2.

In studies on CCA, the ethanol extract of the rhizome of *A. lancea* demonstrated superior selective cytotoxicity compared to the control drug 5-fluorouracil [88]. Its ability to inhibit tumor growth and metastasis, as well as to prolong survival, was further confirmed in both CCA-xenograft nude mouse model [89] and *Opisthorchis viverrini*/dimethylnitrosamine-induced CCA hamster model [90]. These promising results have facilitated the advancement of its standardized capsule formulation, CMC-*A. lancea*, into clinical research [91–93]. The primary active constituents, *β*-eudesmol and atractylodin, exert anti-CCA effects by inducing G1 phase cell cycle arrest, activating caspase-3/7-mediated apoptosis [6], and suppressing multiple key signaling pathways such as HO-1, STAT1/3, and NF-κB [94, 95]. Additionally, *β*-eudesmol has been shown to enhance the efficacy of 5-fluorouracil and doxorubicin against NQO1-high CCA cells [96]. Meanwhile, atractylodin inhibits tumors by modulating the PI3K/AKT/mTOR and p38MAPK signaling pathways [97].

In studies on gastric cancer, the PE fraction of *A. lancea* rhizome extract has been shown to significantly inhibit the growth of human gastric cancer cell lines BGC-823 and SGC-7901 [98]. Among its major constituents, AT-I and AT-II regulate proliferation and apoptosis in gastric cancer cells by suppressing Notch signaling and modulating the Akt/ERK pathway, respectively [99, 100]. AT-III has been shown to ameliorate gastric precancerous lesions and suppress angiogenesis, effects that may be contributed by the downregulation of angiogenesis-associated HIF-1α, VEGF-A, and DLL4 expression [101]. In studies on lung cancer, AT-I has been found to suppress tumor cell proliferation through downregulation of PDK1 gene expression [102], whereas AT-II inhibits cancer metastasis both in vitro and in vivo by preventing macrophage M2-like polarization [103]. In liver cancer studies, AT-II has been shown to modulate proliferation, ferroptosis, and immune evasion of HCC cells via suppression of the TRAF6/NF-κB pathway [104]. AT-III suppresses tumor growth and induces apoptosis by upregulating miR-195-5p and downregulating FGFR1 expression [105]. Atractylon triggers mitochondrial apoptosis and inhibits EMT [106]. Additionally, atractylodin potentially induces ferroptosis in HCC cells by inhibiting GPX4 and FTL protein expression while upregulating ACSL4 and TFR1 levels [107]. In breast cancer research, AT-I and AT-II suppress breast carcinogenesis through inhibition of the TLR4-mediated NF-κB pathway and activation of the Nrf2-ARE signaling axis, respectively [108, 109]. Atractylodin induces both apoptosis and autophagy in MCF-7 cells by promoting oxidative stress and modulating the expression of key proteins regulating apoptotic and autophagic processes [110]. Regarding triple-negative breast cancer, the crude extract of *A. lancea* exhibits notable anti- triple-negative breast cancer activity by modulating EMT markers, suppressing MMP-9 activity, and inhibiting NF-κB signaling [111]. Furthermore, AT-I inhibits proliferation, migration, invasion, and glycolysis in triple-negative breast cancer cells through downregulation of TPI1 and GPI expression [112], and it also enhances the chemosensitivity of triple-negative breast cancer cells to paclitaxel by blocking CTGF expression and inhibiting fibroblast activation [113].

Recent studies have revealed that in castration-resistant prostate cancer, AT-I suppresses cell proliferation and induces apoptosis by targeting KIF15 to mediate the ubiquitin-dependent degradation of AR/AR-V7. Furthermore, when combined with enzalutamide, AT-I exhibits synergistic anti-tumor activity and reverses drug resistance [114]. In clear cell renal cell carcinoma, AT-I upregulates ATP6V0D2 to promote the autophagic degradation of EPAS1/HIF-2α, thereby inhibiting angiogenesis and reversing sunitinib resistance [115]. In endometrial cancer, AT-II exerts its anticancer effects by targeting PADI3 to inhibit the ERK signaling pathway, thereby modulating the expression of proteins associated with glycolysis and apoptosis [116]. In glioblastoma, it induces cell cycle arrest through the MAPK pathway [117], while in colorectal cancer, the compound suppresses tumor progression via the NF-κB p65/PD-L1 signaling axis [118]. Meanwhile, AT-III inhibits the migration and invasion of cervical cancer cells by regulating IGF2BP3 through ETV5 [119].

### Anti-inflammatory effects

In TCM theory, inflammation is often associated with the pathological factors of "dampness-heat." Plants of the *Atractylodes* genus, known for their functions of "drying dampness and strengthening the spleen," are believed to alleviate inflammatory symptoms caused by dampness-heat by regulating spleen-stomach function and resolving dampness [17]. Consequently, they demonstrate potential value in the treatment of inflammation-related diseases Table 3.

In studies on gastroenteritis, the ethanol extract of *A. chinensis* rhizomes alleviates gastric tissue damage and exerts anti-gastritis effects by suppressing the Akt/IκBα/NF-κB signaling pathway [120]. Atractylodin reduces the levels of pro-inflammatory cytokines TNF-α, IL-1β, and IL-6, and suppresses the expression of iNOS and NF-κB in jejunal tissue, thereby ameliorating jejunal inflammation [7]. AT-I modulates the SPHK1/PI3K/AKT axis to inhibit inflammatory responses and targets both SPHK1 and B4GALT2 to regulate the fructose/galactose metabolic pathway, subsequently influencing gut microbiota composition [121]. Additionally, it alleviates DSS-induced ulcerative colitis symptoms by inhibiting S100A8/A9 transcription and regulating the AMPK/mTOR pathway [122]. AT-III mitigates 2,4,6-trinitrobenzenesulfonic acid-induced acute colitis by modulating oxidative stress via FPR1 and Nrf2 pathways and promoting gut microbiota homeostasis [123]. Furthermore, it exerts a protective effect against ulcerative colitis by activating the AMPK/SIRT1/PGC-1α pathway and improving mitochondrial function [124]. Hinesol, another sesquiterpenoid, ameliorates DSS-induced ulcerative colitis through inhibition of Src-mediated NF-κB and chemokine signaling pathways [125].

In studies on vascular inflammation, AT-I effectively suppresses angiogenesis by downregulating multiple factors involved in inflammation and vascular processes, including NO, TNF-α, IL-1β, IL-6, VEGF, and PlGF. In oxidized low-density lipoprotein-induced VSMCs [126], AT-I exhibits anti-atherosclerotic effects by inhibiting VSMC proliferation and migration, preventing macrophage foam cell formation, and suppressing the p38 MAPK/NF-κB pathway [127].

In studies on neuroinflammation, ALR-ELNs downregulate TLR4 expression in BV-2 microglial cells, thereby suppressing the release of inflammatory mediators [128]. AT-III ameliorates ischemic injury and neuroinflammation by inhibiting JAK2/STAT3/Drp1-dependent mitochondrial fission in microglia [129]. It also downregulates TLR4 expression, leading to suppression of p38 MAPK and JNK pathway activation, as well as reduced production of pro-inflammatory factors and enzymes upon LPS stimulation [130]. In a rat model of spinal cord injury, AT-III not only promoted histological and functional recovery but also induced a shift in microglia/macrophage polarization from the M1 to the M2 phenotype, thereby mitigating neuroinflammatory responses [25].

Furthermore, AT-III has been shown to mitigate the progression of osteoarthritis in chondrocytes by attenuating NF-κB signaling activation and delaying cellular senescence, thereby slowing disease advancement [131]. In both IL-4-induced human bronchial epithelial cells and ovalbumin-induced asthmatic mouse models, AT-III suppressed NLRP3 inflammasome activation and modulated the Th1/Th2 balance, suggesting its potential therapeutic value for pediatric asthma [132].

### Neuroprotective effects

Plants of the genus *Atractylodes* and their active constituents demonstrate multi-target neuroprotective potential in neurodegenerative diseases, depression, and anxiety disorders through mechanisms such as anti-inflammatory, anti-apoptotic, and antioxidant effects, autophagy regulation, and modulation of multiple neural signaling pathways [12], Table 4. 

In terms of cognitive improvement, the aqueous extract of *A. macrocephala* not only enhances the activity of antioxidant enzymes in brain tissue [8] but also upregulates the expression of memory-related proteins in the hippocampus, thereby significantly improving learning and memory abilities in D-galactose-induced brain aging mice [133]. Biatractylolide alleviates memory impairment by reducing ROS production, inhibiting AChE activity, and upregulating synaptic-related protein expression [134]. AT-III not only mitigates neuronal damage and reduces Tau protein phosphorylation by modulating multiple signaling pathways, including PI3K/Akt/mTOR and PI3K/AKT/GSK3β [135, 136], but also improves cognitive function by suppressing ROS generation and restoring p-PKC expression [137]. Furthermore, atractylon suppresses CIH-induced M1 microglial activation through an SIRT3-dependent mechanism, thereby preventing sleep-disordered breathing-associated cognitive decline [138].

Targeting glutamate-induced excitotoxicity—a key pathological mechanism in multiple neurological disorders, active constituents of *Atractylodes* species demonstrate remarkable anti-apoptotic efficacy. Biatractylolide protects neuronal cells from glutamate-induced damage via the PI3K-Akt-GSK3β pathway [139]. AT-I inhibits apoptosis induced by MPP [140], while AT-III counteracts glutamate-induced neuronal apoptosis through suppression of caspase signaling [141].

In the pathogenesis of alzheimer′s disease, cholinergic dysfunction and Aβ deposition represent two widely accepted core hypotheses. Addressing the former, biatractylolide-II exhibits AChE inhibitory activity and functions as a dual-site binding inhibitor, simultaneously targeting both the catalytic active site and the peripheral anionic site of the enzyme, while also demonstrating favorable blood–brain barrier permeability [142]. On the other hand, Aβ peptides derived from amyloid precursor protein (APP) cleavage exert neurotoxicity upon abnormal aggregation, representing another core mechanism in alzheimer′s disease pathogenesis. In this context, the ethanol extract of *A. macrocephala* activates the LKB1-AMPK-TFEB pathway, enhances lysosomal function and autophagic flux, and thereby promotes APP degradation while reducing Aβ production [143]. Biatractylolide reduces hippocampal neuronal apoptosis and ameliorates Aβ-induced cognitive impairment [144]. A chemically modified AT derivative not only potently inhibits LSD1 (IC₅₀ = 0.8 μM) and Aβ aggregation, but also alleviates neuroinflammation, reduces Aβ deposition in the hippocampus, and improves cognitive function in APP/PS1 transgenic mouse models [145]. Similarly, AT-III restores autophagic function by modulating the YY1-TFEB axis and reduces Aβ plaque burden in *Caenorhabditis elegans*, cellular models, and APP/PS1 mice [146]. Furthermore, in Parkinson's disease research, AT-I has demonstrated notable anti-neuroinflammatory effects and exhibited therapeutic potential in LPS-induced parkinson's disease models, suggesting its promise as a candidate for parkinson's disease treatment [147].

In antidepressant and anxiolytic research, AT-I exerts antidepressant-like effects in a CUMS-induced mouse model of depression by inhibiting NLRP3 inflammasome activation and reducing IL-1β production [148]. AT-III demonstrates significant antidepressant and anxiolytic efficacy in both cellular and animal models through suppression of intracellular Ca^2^⁺ overload, the mitochondrial apoptotic pathway, and modulation of the MAPK/NF-κB inflammatory axis [149]. Atractylodin not only modulates the ferroptosis pathway but also promotes the expression of neurotrophic factors, thereby conferring multi-target neuroprotective and antidepressant activities in depression models [150].

### Gastrointestinal function-improving effects

According to TCM theory, plants of the genus *Atractylodes* are attributed with the function of "strengthening the spleen," which represents a comprehensive multi-system regulatory action. This concept corresponds primarily to the digestive and absorptive systems in modern medicine, encompassing the regulation of gastrointestinal motility, alleviation of intestinal functional disorders, maintenance of intestinal barrier integrity, and modulation of gut microbiota balance, thereby helping to relieve symptoms such as constipation and diarrhea. Furthermore, their dampness-drying contribute to eliminating excess dampness from the body and alleviating gastrointestinal discomfort caused by dampness obstructing the middle energizer [12, 17], Table 5.

In studies on gastrointestinal motility regulation, the extract of *A. lancea* and *β*-eudesmol may promote gastric emptying and small intestinal motility via inhibition of dopamine D_2_ and 5-HT_3_ receptors [151]. In addition, the ethanol extract of *A. macrocephala* effectively suppresses spontaneous colonic contractions and the migrating motor complex in humans by inhibiting the pacemaker activity of interstitial cells of Cajal and modulating the cAMP/ATP-sensitive K⁺ channel pathway. It also ameliorates intestinal hypermotility in mouse models, demonstrating clear potential as a gastrointestinal motility modulator [9].

In addressing intestinal dysfunction such as diarrhea and constipation, the aqueous extract of bran-fried rhizomes of *A. macrocephala* improves gastrointestinal health and ameliorates pregnancy status and outcomes in spleen deficiency diarrhea pregnant mice through multiple pathways. These include modulating gastrointestinal motility, elevating gastrointestinal hormone levels, protecting intestinal barrier function, and regulating fluid metabolism balance [152]. AT-III inhibits pyrotinib-induced chloride secretion through the AMPK/CFTR signaling pathway, alleviates diarrhea by modulating intestinal flora and increasing lithocholic acid, without compromising the anticancer efficacy of pyrotinib [153]. In constipation models, the aqueous extract of *A. macrocephala* ameliorates intestinal function and pathological morphology in spleen deficiency constipation rats by modulating gut microbiota composition and bile acid metabolism, with the TGR5 receptor identified as a key target [154]. Furthermore, *A. macrocephala* improves gastrointestinal function and neurotransmitter levels in loperamide-induced constipated rats through regulation of the tryptophan metabolic pathway, with medium and high doses exhibiting particularly significant effects [155].

In studies on gastric mucosal protection and ulcer treatment, studies have confirmed that bran-processed *A. lancea* is more effective than crude *A. lancea* in alleviating acetic acid-induced gastric ulcers. The underlying mechanism is closely associated with the modulation of anti-inflammatory factors and upregulation of gastroprotective factors EGF and TFF2 [156]. Further studies have revealed that the active constituent atractyloside A modulates gastrointestinal hormones and tight junction protein expression, while also protecting the intestinal mucosal barrier through suppression of the p38 MAPK pathway [157]. Furthermore, AT-I alleviates gastric ulcer inflammation by inhibiting the NLRP3 inflammasome pathway [158], while AT-III has been identified as the primary gastroprotective component of *A. ovata* against ethanol-induced acute gastric ulcers, acting through suppression of MMP-2 and MMP-9 pathways [159]. It is noteworthy that recent studies have for the first time revealed anti-*Helicobacter pylori* activity in multiple constituents from *A. macrocephala*. Among them, AT-I exhibited potent inhibition against three *H. pylori* strains comparable to metronidazole, while AT-III showed selective activity against specific strains. In contrast, polyacetylene constituents demonstrated relatively weak effects [160].

In irritable bowel syndrome and other intestinal mucosal disorders, the ethanol extract of *A. macrocephala* alleviates irritable bowel syndrome symptoms by modulating TRPV1, NaV1.5, and NaV1.7 channels [161]. AT-I ameliorates oxidative stress-induced colonic mucosal epithelial dysfunction through the miR-34a-5p–LDHA–glucose metabolism axis [162], stimulates intestinal epithelial cell migration and proliferation via polyamine-mediated Ca^2^⁺ signaling, demonstrating therapeutic potential for intestinal mucosal injury [163]. Furthermore, this compound may modulate gut microbiota by regulating the TLR4/MyD88/NF-κB signaling pathway [164].

### Hepatoprotective effects

Recent studies have revealed that AT-III targets ASCT2 and modulates the IL-1α/NF-κB feedback pathway involved in glutaminolysis, thereby inducing HSC senescence while ameliorating the SASP [10]. Furthermore, AT-III suppresses SASP production and its paracrine effects by inhibiting the cGAS/NF-κB signaling pathway [165]. More importantly, this compound also exerts anti-fibrotic effects through multi-pathway inhibition of the PI3K/AKT pathway and glutamine metabolism [166]. Consequently, AT-III achieves a dual therapeutic effect of "inducing senescence" and "suppressing inflammation," offering novel insights into liver fibrosis treatment and demonstrating potential as a therapeutic agent (Tables 1, 2, 3, 4, 5, 6, 7, 8).

**Table 1:** Isolation and identification of compounds from the plants of the genus *Atractylodes*

No	Name	Species	Molecular weight	Molecular formula	References
1	4*α*,7*α*-Epoxyguaiane-10*α*,11-diol	*A. lancea*	254.19	C_15_H_26_O_3_	[38]
2	7*α*,10*α*-Epoxyguaiane-4*α*,11-diol	*A. lancea*	254.19	C_15_H_26_O_3_	[38]
3	10*β*,11*β*-Epoxyguaiane-1*α*, 4*α*-diol	*A. lancea*	254.19	C_15_H_26_O_3_	[38]
4	10*β*,11*β*-Epoxyguaiane-1*α*,4*α*,7*α*-triol	*A. lancea*	270.18	C_15_H_26_O_4_	[38]
5	1-Patchoulene-4*α*,7*α*-diol	*A. lancea*	236.18	C_15_H_24_O_2_	[38]
6	Dehydrocostus lactone	*A. japonica*	230.13	C_15_H_18_O_2_	[62]
7	Atractyloside A	*A. lancea*	448.23	C_21_H_36_O_10_	[55]
8	Atractyloside B	*A. lancea*	450.25	C_21_H_38_O_10_	[55]
9	(1*S*,4*S*,5*S*,7*R*,10*R*)−10,11,14-Trihydroxyguai-3-one 11-*O*-*β*-D-glucopyranoside	*A. lancea*	432.24	C_21_H_36_O_9_	[55]
10	(1*S*,4*S*,5*R*,7*R*,10*R*)−11,14-Dihydroxyguai-3-one11-*O*-*β*-D-glucopyranoside	*A. lancea*	432.24	C_21_H_36_O_9_	[55]
11	(1*S*,5*R*,7*R*,10*R*)-Secoatractylolactone11-*O*-*β*-D-glucopyranoside	*A. lancea*	446.22	C_21_H_34_O_10_	[55]
12	Atractyloside A 14-*O*-*β*-D-fructofuranoside	*A. lancea*	610.28	C_27_H_46_O_15_	[55]
13	10-Epi-atractylosideA	*A. lancea*	448.23	C_21_H_36_O_10_	[55]
14	(1*S*,4*S*,5*S*,7*R*,10*R*)−10.11,14-Trihydroxyguai-3-one 11-*O*-*β*-D-glucopyranoside	*A. lancea*	416.24	C_21_H_36_O_8_	[55]
15	(1*S*,4*S*,5*S*,7*R*,10*S*)−10.11,14-Trihydroxyguai-3-one11-*O*-*β*-D-glucopyranoside	*A. lancea*	416.24	C_21_H_36_O_8_	[55]
16	(1*S*,5*R*,7*R*,10*R*)-Secoatractylolactone-11-*O*-*β*-D-glucopyranoside	*A. lancea*	458.25	C_23_H_38_O_9_	[55]
17	3,4,11,14-Tetrahydroxyguai-9-en-11-*O*-*β*-D-glucopyranoside	*A. lancea*	432.24	C_21_H_36_O_9_	[63]
18	(3*R*,4*R*,7*R*,10*R*)−2-Hydroxypancherione-11-*O*-*β*-D-glucopyranoside	*A. lancea*	414.23	C_21_H_34_O_8_	[40]
19	(1*S*,7*R*,10*R*)−11,15-Dihydroxy-4-guaien-3-one 11-*O*-*β*-D-glucopyranoside	*A. lancea*	414.23	C_21_H_34_O_8_	[5]
20	(1*R*,7*R*,10*S*)−10,11-Dihydroxy-4-guaien-3-one 11-*O*-*β*-D-glucopyranoside	*A. lancea*	414.23	C_21_H_34_O_8_	[41]
21	(1*S*,4*S*,5*R*,7*R*)−4,11,14-Tri-hydroxy-guaia-9-en-3-one-11-*O*-*β*-D-glucopyranoside	*A. lancea*	430.22	C_21_H_34_O_9_	[63]
22	(1*S*,4*S*,5*R*,7*R*,10*S*)−4,11,14-Trihydroxy-guai-3-one-11-*O*-*β*-D-glucopyranoside	*A. lancea*	432.24	C_21_H_36_O_9_	[63]
23	(1*R*,5*S*,7*R*,10*R*)-Secoatractylo-*δ*-lactone-11-*O*-*β*-D-glucopyranoside	*A. lancea*	430.22	C_21_H_34_O_9_	[63]
24	Dihydroxy-9-guaine-3-one-11-*O*-*β*-D-glucopyranoside	*A. lancea*	414.21	C_21_H_34_O_8_	[64]
25	(1*S*,4*S*,5*S*,7*R*)−4,13-Dihydroxyguaia-10-ene	*A. lancea*	234.15	C_15_H_26_O_2_	[42]
26	(4*S*,5*R*,7*S*)−5-Hydroxyguaia-1(10),13-dien-9-one	*A. lancea*	234.15	C_15_H_26_O_2_	[42]
27	Magnodelavin C	*A. lancea*	220.18	C_15_H_24_O	[42]
28	Atractylol A	*A. lancea*	256.20	C_15_H_28_O_3_	[59]
29	Atrchiterpene D	*A. chinensis*	272.20	C_15_H_28_O_4_	[65]
30	(4*S*,5*S*)-Atractylmacrene A	*A. macrocephala*	220.17	C_15_H_24_O	[44]
31	(4*R*,5*R*)-Atractylmacrene A	*A. macrocephala*	220.17	C_15_H_24_O	[44]
32	(1*S*,4*S*,5*S*)-Atractylmacrene B	*A. macrocephala*	220.17	C_15_H_24_O	[44]
33	(1*R*,4*R*,5*R*)-Atractylmacrene B	*A. macrocephala*	220.17	C_15_H_24_O	[44]
34	Guaianoside I	*A. japonica*	474.28	C_23_H_38_O_10_	[60]
35	Guaianoside II	*A. japonica*	416.27	C_21_H_36_O_8_	[60]
36	(1*S*,5*R*,7*R*,10*R*)-Seco-atractylolactone	*A. lancea*	284.16	C_15_H_24_O_5_	[64]
37	(5*R*,7*R*,10*S*)−3-*O*-*β*-D-Glucopyranosylisopterocarpolone-11-*O*-*β*-D-apiofur-anosyl-(1 → 6)-*β*-D-glucopyranoside	*A. lancea*	708.32	C_32_H_52_O_17_	[41]
38	Eudesm-4 (15)-ene-7*α*,11-diol	*A. lancea*	254.19	C_15_H_26_O_3_	[38]
39	Eudesm-4 (15),7-diene-9*α*,11-diol	*A. lancea*	236.18	C_15_H_24_O_2_	[38]
40	Eudesm-4 (15),7-diene-11-ol-9-one	*A. lancea*	234.16	C_15_H_22_O_2_	[38]
41	*β*-Eudesmol	*A. lancea*	238.19	C_15_H_26_O_2_	[46]
42	14-Hydroxy-isopterocarpolone	*A. lancea*	252.17	C_15_H_24_O_3_	[59]
43	Kudtdiol	*A. lancea*	238.19	C_15_H_26_O_2_	[59]
44	(11*R*)−2,11,12-Trihydroxy-*β*-selinene	*A. lancea*	254.19	C_15_H_26_O_3_	[59]
45	2,11,13-Trihydroxy-*β*-selinene	*A. lancea*	254.19	C_15_H_26_O_3_	[59]
46	3*α*-Hydroxy-pterocarpol	*A. lancea*	254.19	C_15_H_26_O_3_	[59]
47	4 (15),11-Eudesmadien	*A. lancea*	204.19	C_15_H_24_	[66]
48	Pterocarpol	*A. lancea*	238.19	C_15_H_26_O_2_	[42]
49	Eudesma-4(14),7(11)-dien-8-one	*A. japonica*	218.17	C_15_H_22_O	[67]
50	(+) Eudesma-4(14),7(11)-dien-8-one	*A. japonica*	218.17	C_15_H_22_O	[67]
51	Atractylon	*A. lancea*	216.15	C_15_H_20_O	[68]
52	AT-I	*A. lancea*	230.13	C_15_H_18_O_2_	[68]
53	AT-II	*A. lancea*	232.15	C_15_H_20_O_2_	[68]
54	AT-III	*A. lancea*	248.14	C_15_H_20_O_3_	[68]
55	Atractylenolide IV	*A. macrocephala*	306.15	C_17_H_22_O_5_	[44]
56	Atractyloside C	*A. lancea*	400.25	C_21_H_36_O_7_	[55]
57	Atractyloside G	*A. lancea*	416.24	C_21_H_36_O_8_	[55]
58	Atractyloside G-2-O-*β*-D-glucopyranoside	*A. lancea*	578.29	C_27_H_46_O_13_	[55]
59	Atractyloside D	*A. lancea*	562.30	C_27_H_46_O_12_	[55]
60	Atractyloside I	*A. lancea*	562.26	C_26_H_42_O_13_	[55]
61	Atractyloside E	*A. lancea*	694.34	C_32_H_54_O_16_	[55]
62	(5*R*,7*R*,10*S*)-Isopterocarpolone-*β*-D-glucopyranoside	*A. lancea*	576.28	C_27_H_44_O_13_	[55]
63	*cis*-Atractyloside I	*A. lancea*	398.23	C_21_H_34_O_7_	[55]
64	Atractyloside G	*A. lancea*	578.29	C_27_H_46_O_13_	[69]
65	(3*S*)−3-Hydroxyatractylenolide III-3-*O*-D-glucopyranoside	*A. japonica*	426.19	C_21_H_30_O_9_	[3]
66	Atractyloside F	*A. lancea*	740.35	C_33_H_56_O_18_	[69]
67	(1*R*,7*R*,10*R*)−1-Hydroxylcarissone-11-*O*-*β*-D-glucopyranoside	*A. lancea*	414.23	C_21_H_34_O_8_	[41]
68	3-Hydroxylisopterocarpolone-3-*O*-*β*-D-glucopyranoside	*A. lancea*	886.40	C_39_H_66_O_22_	[39]
69	(5*R*,7*R*,10*S*)−3-*O*-*β*-D-glucopyranosylisopterocarpolone-11-*O*-*β*-D-apiofuranosyl-(1 → 6)-*β*-D-glucopyranoside	*A. lancea*	708.32	C_32_H_52_O_17_	[41]
70	6''-*O*-Acetylatractyloside I	*A. lancea*	618.29	C_29_H_46_O_14_	[39]
71	(5*R*,7*R*,10*S*)−6'-*O*-Acetylatractyloside I	*A. lancea*	618.29	C_29_H_46_O_14_	[39]
72	Isopterocarpolone-11-*O*-*β*-D-apiofuranosyl-(1 → 6)-*β*-D-glucopyranoside	*A. lancea*	530.27	C_26_H_42_O_11_	[39]
73	(5*R*,7*R*,10*S*)−14-Hydroxylisopterocarpolone-11-*O*-*β*-D-glueopyranoside	*A. lancea*	414.23	C_21_H_34_O_8_	[1]
74	(5*R*,7*R*,10*S*)−14-Carboxylisopterocarpolone-11-*O*-*β*-D-glucopyranoside	*A. lancea*	428.20	C_21_H_32_O_9_	[41]
75	(2*S*,7*R*,10*S*)−2-Hydroxylcarissone-11-*O*-*β*-D-glucopyranoside	*A. lancea*	414.23	C_21_H_34_O_8_	[39]
76	(2*R*,7*R*,10*S*)−2-Hydroxylcarissone-11-*O*-*β*-D-glucopyranoside	*A. lancea*	414.23	C_21_H_34_O_8_	[39]
77	Atramacrolode A	*A. macrocephala*	252.17	C_15_H_24_O_3_	[70]
78	Atramacrolode B	*A. macrocephala*	234.16	C_15_H_22_O_2_	[70]
79	Atramacrolode C	*A. macrocephala*	256.18	C_18_H_24_O	[70]
80	Atramacrolode D	*A. macrocephala*	362.25	C_22_H_34_O_4_	[70]
81	Eudesm-4(15)-ene-7,11-diol	*A. macrocephala*	238.19	C_15_H_26_O_2_	[70]
82	Clasamane A	*A. macrocephala*	276.17	C_17_H_24_O_3_	[70]
83	Atractylenolide VII	*A. macrocephala*	262.19	C_17_H_26_O_2_	[70]
84	9*α*-Hydroxyasterolide	*A. macrocephala*	248.14	C_15_H_20_O_3_	[70]
85	8*β*-Methoxyatractylenolide	*A. macrocephala*	262.16	C_16_H_22_O_3_	[70]
86	Atrchiterpene A	*A. chinensis*	315.18	C_19_H_25_NO_3_	[65]
87	Atrchiterpene B	*A. chinensis*	260.14	C_16_H_20_O_3_	[65]
88	Eudesmane-4*α*,11,15-triol	*A. chinensis*	256.20	C_15_H_28_O_3_	[65]
89	15-Acetoxy-eudesma-3,7(11),8-trien-12,8-olide	*A. lancea*	288.14	C_17_H_20_O_4_	[43]
90	3*β*-Acetoxyl-atractylenolide I	*A. lancea*	302.15	C_18_H_22_O_4_	[43]
91	7*α*-Hydroxy-selina-4(15),11(12)-dien-8-one	*A. lancea*	234.16	C_15_H_22_O_2_	[43]
92	Macrophyllilactone E	*A. lancea*	248.14	C_15_H_20_O_3_	[43]
93	Septuplinolide	*A. lancea*	250.16	C_15_H_22_O_3_	[43]
94	Atractyloside I	*A. lancea*	576.28	C_27_H_44_O_13_	[63]
95	(−)-Eudesma-4(15)-ene-2*β*,11-diol	*A. lancea*	238.19	C_15_H_26_O_2_	[42]
96	1*β*-Hydroxy-*β*-eudesmol	*A. lancea*	238.19	C_15_H_26_O_2_	[42]
97	3-Eudesmene-l*β*,11-diol	*A. lancea*	238.19	C_15_H_26_O_2_	[42]
98	Cryptomeridiol	*A. lancea*	240.21	C_15_H_28_O_2_	[42]
99	*β*-Eudesmol	*A. lancea*	238.19	C_15_H_26_O_2_	[42]
100	Eudesm-4(14)-ene-3*α*,11-diol	*A. lancea*	238.19	C_15_H_26_O_2_	[42]
101	7*α*,11-Dihydroxycadin-10(14)-ene	*A. lancea*	238.19	C_15_H_26_O_2_	[42]
102	(1*S*,5*R*,10*S*)-Atractylmacrene C	*A. macrocephala*	220.18	C_15_H_24_O	[44]
103	(1*R*,5*S*,10*R*)-Atractylmacrene C	*A. macrocephala*	220.18	C_15_H_24_O	[44]
104	4*R*,5*R*,8*S*,9*S*-diepoxyatractylenolide II	*A. macrocephala*	262.12	C_15_H_18_O_4_	[44]
105	4-Oxo-8*S*,9*S*-epoxylatractylenolide II	*A. macrocephala*	248.10	C_14_H_16_O_4_	[44]
106	8*S*,9*S*-Epoxylatractylenolide II	*A. macrocephala*	246.13	C_15_H_18_O_3_	[44]
107	Juniper camphor	*A. macrocephala*	222.20	C_15_H_26_O	[44]
108	4*R*,15-Epoxy-8*β*-hydroxylatractylenolide II	*A. macrocephala*	264.14	C_15_H_20_O_4_	[44]
109	8*β*,9*α*-Dihydroxylatractylenolide II	*A. macrocephala*	264.14	C_15_H_20_O_4_	[44]
110	Atractylenolactam	*A. macrocephala*	229.15	C_15_H_29_NO	[44]
111	Selina-4(14),7,11-trien-9-ol	*A. macrocephala*	218.17	C_15_H_22_O	[71]
112	Selina-4(14),11-dien-7-ol	*A. macrocephala*	220.18	C_15_H_24_O	[71]
113	Eudesm-4(15),7-diene-3*α*,9*β*,11-triol	*A. macrocephala*	252.17	C_15_H_24_O_3_	[72]
114	Eudesm-4(15),7-diene-1*β*,3*α*,9*β*,11-tetraol	*A. macrocephala*	268.17	C_15_H_24_O_4_	[72]
115	Atramacronoid S	*A. macrocephala*	339.18	C_21_H_25_NO_3_	[58]
116	Atramacronoid T	*A. macrocephala*	359.25	C_21_H_25_NO_3_	[58]
117	Atramacronoid U	*A. macrocephala*	246.13	C_15_H_18_O_3_	[58]
118	Atramacronoid V	*A. macrocephala*	246.13	C_15_H_18_O_3_	[58]
119	8*α*-Methoxy-epiasterolid	*A. macrocephala*	262.16	C_16_H_22_O_3_	[45]
120	3*β*-Acetoxyl-8-epiasterolid	*A. macrocephala*	290.16	C_17_H_22_O_4_	[45]
121	3*β*-Acetoxyl atractylenolide I	*A. macrocephala*	290.15	C_17_H_22_O_4_	[45]
122	Isoatractylenolide I	*A. japonica*	232.15	C_15_H_20_O_2_	[60]
123	Cryptomeridiol	*A. japonica*	240.21	C_15_H_28_O_2_	[60]
124	(4*R*,5*R*,7*R*,10*R*)−4-Hydroxy-eudesma-2,11-dien-1-one	*A. japonica*	234.16	C_15_H_22_O_2_	[60]
125	Carainterol A	*A. japonica*	238.19	C_15_H_26_O_2_	[60]
126	6*α*-Hydroxy-4(15)-eudesmen-1-one	*A. japonica*	236.18	C_15_H_24_O_2_	[60]
127	Biepiatractylenolide	*A. macrocephala*	462.28	C_30_H_38_O_4_	[44]
128	Biatractylenolide	*A. macrocephala*	462.28	C_30_H_38_O_4_	[44]
129	Biepiasreorlid II	*A. macrocephala*	461.27	C_30_H_36_O_4_	[45]
130	Biepiasreorlid	*A. macrocephala*	462.28	C_30_H_38_O_4_	[45]
131	Biatractylolide	*A. macrocephala*	462.28	C_30_H_38_O_4_	[45]
132	Biatractylonoid A	*A. macrocephala*	496.28	C_30_H_40_O_6_	[13]
133	Biatractylonoid B	*A. macrocephala*	496.28	C_30_H_40_O_6_	[13]
134	Biatractylonoid C	*A. macrocephala*	496.28	C_30_H_40_O_6_	[13]
135	Biatractylonoid D	*A. macrocephala*	476.29	C_31_H_40_O_4_	[13]
136	Biatractylonoid E	*A. macrocephala*	512.29	C_34_H_40_O_4_	[13]
137	(3*S*,4*R*,5*S*,7*R*)−13-Hydroxylhinesolone-11-*O*-*β*-D-glucopyranoside	*A. lancea*	414.23	C_21_H_34_O_8_	[5]
138	Hinesol	*A. lancea*	222.20	C_15_H_26_O	[42]
139	Hinesolone	*A. lancea*	236.18	C_15_H_24_O_2_	[42]
140	2-Oxo-12-hydroxy-hinesol	*A. lancea*	252.17	C_15_H_24_O_3_	[40]
141	2-Oxo-15-hydroxy-hinesol	*A. lancea*	252.17	C_15_H_24_O_3_	[40]
142	(7*R*)−3,4-Dehydrohinesolone-11-*O*-*β*-D-glucopyranoside	*A. lancea*	396.21	C_21_H_32_O_7_	[40]
143	(7*R*)−3,4-dehydrohinesolone-11-*O*-*β*-D-apiofuranosyl-(1 → 6)-*β*-D-glucopyranoside	*A. lancea*	528.60	C_26_H_40_O_11_	[40]
144	(5*R*,7*R*)−14-Hydroxy-3,4-dehydrohinesolone-11-*O*-*β*-D-glueopyranoside	*A. lancea*	412.21	C_21_H_32_O_8_	[40]
145	(5*R*,7*R*)−14-Hydroxy-3,4-dehydrohinesolone-11-*O*-*β*-D-apiofuranosyl-(1 → 6)-*β*-D-glucopyranoside	*A. lancea*	544.25	C_26_H_40_O_12_	[40]
146	(5*R*,7*R*)−14-Hydroxy-3,4-dehydrohinesolone-14-*O*-*β*-D-xylopyranoside	*A. lancea*	382.20	C_20_H_30_O_7_	[40]
147	(4*S*,5*S*,7*R*)−15-Hydroxylhinesolone-15-*O*-*β*-D-xylopyranoside	*A. lancea*	384.21	C_20_H_32_O_7_	[40]
148	(4*S*,5*S*,7*R*)−14-Hydroxylhinesolone-14-*O*-*β*-D-xylopyranoside	*A. lancea*	384.21	C_20_H_32_O_7_	[40]
149	(+)-Hinesol	*A. lancea*	236.18	C_15_H_24_O_2_	[43]
150	(1*R*,4*S*,5*S*,7*R*)−1,13-Dihydroxyspirovetiv-10-ene	*A. lancea*	252.17	C_15_H_24_O_3_	[42]
151	(1*S*,4*S*,5*S*,7*R*)−1,13-Dihydroxyspirovetiv-10-ene	*A. lancea*	252.17	C_15_H_24_O_3_	[42]
152	(4*S*,5*S*,7*R*)−13-Hydroxyspirovetiv-10-en-1-one	*A. lancea*	250.16	C_15_H_22_O_3_	[42]
153	2*β*-Hydroxyhinesol	*A. lancea*	238.19	C_15_H_26_O_2_	[42]
154	2*α*-Hydroxyhinesol	*A. lancea*	238.19	C_15_H_26_O_2_	[42]
155	11-Hydroxyhinesol	*A. lancea*	238.19	C_15_H_26_O_2_	[42]
156	(4*R*, 5*S*, 7*R*)-Hinesolone-11-*O*-*β*-D-glucopyranoside	*A. lancea*	398.23	C_21_H_34_O_7_	[73]
157	(3*S*,4*R*,5*R*,7*R*)−3,11-Dihydroxy-11,12-dihydronootkatone-11-*O*-*β*-D-glucopyranoside	*A. lancea*	414.23	C_21_H_34_O_8_	[1]
158	(3*S*,4*R*,5*S*,7*R*)−3,4,11-Trihydroxy-11,12-dihydronootkatone-11-*O*-*β*-D-glucopyranoside	*A. lancea*	430.22	C_21_H_34_O_9_	[1]
159	Germacrene B	*A. lancea*	204.19	C_15_H_24_	[43]
160	Atralanol A	*A. lancea*	250.19	C_16_H_26_O_2_	[42]
161	Atralanol B	*A. lancea*	252.17	C_15_H_24_O_3_	[42]
162	10*β*-Hydroxyisodauc-6-en-14-al	*A. japonica*	236.18	C_15_H_24_O_2_	[60]
163	Atramacronoid W	*A. macrocephala*	306.18	C_18_H_26_O_4_	[58]
164	*α*-Bisabolol	*A. lancea*	222.20	C_15_H_26_O	[74]
165	Stigmasterol-3-*O*-*β*-D-glucopyranoside	*A. lancea*	560.41	C_34_H_56_O_6_	[46]
166	Daucosterol	*A. lancea*	562.42	C_34_H_58_O_6_	[46]
167	Stigmasterol	*A. lancea*	398.35	C_28_H_46_O	[46]
168	*β*-Sitosterol	*A. lancea*	400.37	C_28_H_48_O	[46]
169	Traxerol acetate	*A. lancea*	466.38	C_32_H_50_O_2_	[46]
170	ϕ-Traxasteryl acetate	*A. lancea*	466.38	C_32_H_50_O_2_	[46]
171	Oleanolic acid	*A. lancea*	456.36	C_30_H_48_O_3_	[75]
172	Atrachinenin D	*A. chinensis*	392.31	C_28_H_40_O	[14]
173	Atrachinenin E	*A. chinensis*	410.28	C_27_H_38_O_3_	[14]
174	Atrachinenin F	*A. chinensis*	410.28	C_27_H_38_O_3_	[14]
175	Atrachinenin G	*A. chinensis*	446.28	C_30_H_38_O_3_	[14]
176	Atrachinenin H	*A. chinensis*	462.28	C_30_H_38_O_4_	[14]
177	Atrachinenin I	*A. chinensis*	490.31	C_32_H_42_O_4_	[14]
178	Atrachinenin J	*A. chinensis*	548.31	C_34_H_44_O_6_	[14]
179	Atrachinenin K	*A. chinensis*	490.31	C_32_H_42_O_4_	[14]
180	Atrachinenin L	*A. chinensis*	480.36	C_32_H_48_O_3_	[14]
181	Atrachinenin M	*A. chinensis*	412.30	C_27_H_40_O_3_	[14]
182	Atrachinenin N	*A. chinensis*	412.30	C_27_H_40_O_3_	[14]
183	Atrachinenin O	*A. chinensis*	428.29	C_27_H_40_O_4_	[14]
184	Atrachinenin P	*A. chinensis*	490.31	C_32_H_42_O_4_	[14]
185	Atrachinenin Q	*A. chinensis*	548.31	C_34_H_44_O_6_	[14]
186	Atrachinenin R	*A. chinensis*	548.31	C_34_H_44_O_6_	[14]
187	Atrachinenin S	*A. chinensis*	344.24	C_22_H_32_O_3_	[14]
188	Atractin A	*A. macrocephala*	512.29	C_34_H_40_O_4_	[49]
189	Atramacronoid A	*A. macrocephala*	354.18	C_22_H_26_O_4_	[50]
190	Atramacronoid B	*A. macrocephala*	340.17	C_21_H_24_O_4_	[50]
191	Atramacronoid C	*A. macrocephala*	354.18	C_22_H_26_O_4_	[50]
192	Atrachinenyne A	*A. chinensis*	468.19	C_30_H_28_O_5_	[48]
193	Atrachinenyne B	*A. chinensis*	436.26	C_28_H_36_O_4_	[48]
194	Atrachinenyne C	*A. chinensis*	518.27	C_32_H_38_O_6_	[48]
195	Atrachinenyne D	*A. chinensis*	504.29	C_32_H_40_O_5_	[48]
196	(+)-Atrachinenin A	*A. chinensis*	408.30	C_28_H_40_O_2_	[47]
197	(−)-Atrachinenin A	*A. chinensis*	408.30	C_28_H_40_O_2_	[47]
198	(+)-Atrachinenin B	*A. chinensis*	426.28	C_27_H_38_O_4_	[47]
199	(−)-Atrachinenin B	*A. chinensis*	426.28	C_27_H_38_O_4_	[47]
200	(+)-Atrachinenin C	*A. chinensis*	410.28	C_27_H_38_O_3_	[47]
201	(−)-Atrachinenin C	*A. chinensis*	410.28	C_27_H_38_O_3_	[47]
202	Atracerpenoid A	*A. macrocephala*	170.09	C_9_H_14_O_3_	[58]
203	Atractylodin	*A. lancea*	182.07	C_13_H_10_O	[76]
204	(1*Z*)-Atractylodin	*A. lancea*	182.07	C_13_H_10_O	[77]
205	9-Nor-atractylodin	*A. lancea*	198.07	C_13_H_10_O_2_	[78]
206	Atractylodinol	*A. lancea*	198.07	C_13_H_10_O_2_	[43]
207	(1*Z*)-Atractylodinol	AC	198.07	C_13_H_10_O_2_	[77]
208	Acetylatractylodinol	*A. lancea*	240.08	C_15_H_12_O_3_	[43]
209	(1*Z*)-Acetylatractylodinol	*A. lancea*	240.08	C_15_H_12_O_3_	[77]
210	(6*E*,12*E*)-Tetradecadiene-8,10-diyne-1,3-diol	*A. chinensis*	218.13	C_14_H_18_O_2_	[75]
211	(6*E*,12*Z*)-Tetradecadiene-8,10-diyne-1,3-diol	*A. chinensis*	218.13	C_14_H_18_O_2_	[79]
212	(6*Z*,12*Z*)-Tetradecadiene-8,10-diyne-1,3-diol	*A. chinensis*	218.13	C_14_H_18_O_2_	[79]
213	(6*E*,12*E*)-Tetradecadiene-8,10-diyne-1,3-diol diacetate	*A. chinensis*	302.15	C_14_H_22_O_4_	[75]
214	(6*E*,12*E*)−3-Acetoxytetradeca-6,12-dien-8,10-diyn-1-ol	*A. chinensis*	260.14	C_16_H_20_O_3_	[75]
215	(6*E*,12*E*)−1-Acetoxytetradeca-6,12-dien-8,10-diyn-3-ol	*A. chinensis*	260.14	C_16_H_20_O_3_	[75]
216	Diacetyl atractylodiol	*A. japonica*	302.15	C_18_H_22_O_4_	[80]
217	(2*E*,8*E*)-Decadiene-4,6-diyne-1,10-diol 1-*O*-*β*-D-glucopyranoside	*A. lancea*	324.12	C_16_H_20_O_7_	[55]
218	(6*E*,12*E*)-Tetradecadiene-8,10-diyne-1,3-diol	*A. lancea*	218.13	C_14_H_18_O_2_	[81]
219	(2*E*,8*Z*)-Deca-2,8-diene-4,6-diyne-1,10-diol-1-*O*-*β*-D-glucopyranoside	*A. lancea*	324.12	C_16_H_20_O_7_	[40]
220	(2*E*,8*Z*)-Deca-2,8-diene-4,6-diyne-1,10-diol-1-*O*-*β*-D-apiofuranosyl-(1 → 6)-*β*-D-glucopyranoside	*A. lancea*	456.16	C_21_H_30_O_11_	[51]
221	(2*E*,8*E*)-Deca-2,8-diene-4,6-diyne-1,10-diol-1-*O*-*β*-D-apiofuranosyl-(1 → 6)-*β*-D-glucopyranoside	*A. lancea*	456.16	C_21_H_28_O_11_	[40]
222	(8*R*,9*S*)−2*E*,10*Z*-Tridecadiene-4,6-diyne-8,9,12,13-triol-9-*O*-*β*-D-glucopyranoside	*A. lancea*	398.16	C_19_H_26_O_9_	[5]
223	(8*R*,9*S*)−2*E*,10*Z*-Tridecadiene-4,6-diyne-8,9,12,13-triol-8-*O*-*β*-D-glucopyranoside	*A. lancea*	398.16	C_19_H_26_O_9_	[5]
224	(8*R*,9*S*)−2*E*,10*Z*-Tridecadiene-4,6-diyne-8,9,12,13-triol	*A. lancea*	236.10	C_13_H_16_O_4_	[5]
225	(2*Z*,8*E*)-Deca-2,8-diene-4,6-diyne-1,10-diol-1-*O*-*β*-D-glucopyranoside	*A. lancea*	324.12	C_16_H_20_O_7_	[40]
226	(2*E*,8*E*,10*R*)-Tridecatriene-4,6-diyne-1,10,11,12,13-pentol-10-*O*-*β*-D-glucopyranoside	*A. lancea*	414.15	C_19_H_26_O_10_	[5]
227	(2*E*,10*S*)-Tridecane-2-ene-4,6-diyne-1,10,13-triol-*O*-*β*-D-xylopyranoside	*A. lancea*	514.24	C_25_H_38_O_11_	[5]
228	(3*R*,8*E*,10*E*)-Tetradecadiene-4,6-diyne-3,12,14-triol-3-*O*-*β*-D-glucopyranoside	*A. lancea*	396.18	C_20_H_28_O_8_	[5]
229	(2*E*,8*E*,12*S*)-Tetradecadiene-4,6-diyne-1,10,14-triol-1-*O*-*β*-D-apiofuranosyl-(1 → 6)-*β*-D-glucopyranoside	*A. lancea*	528.22	C_25_H_36_O_12_	[5]
230	(1,3*Z*,11*E*)-Tridecatriene-7,9-diyne-5-hydroxyl-6-*O*-*β*-D-glucopyranoside	*A. lancea*	364.15	C_19_H_24_O_7_	[82]
231	Atractyloyne	AC	316.17	C_19_H_24_O_4_	[83]
232	(4*E*,6*E*,12*E*)-Tetradecatriene-8,10-diyne-1,3-diyl diacetate	*A. lancea*	300.14	C_18_H_20_O_4_	[77]
233	(4*E*,6*E*,12*E*)-Tetradecatrien-8,10-diyn-1-ol	*A. lancea*	200.12	C_14_H_16_O	[75]
234	(4*E*,6*E*,12*E*)−3-Isovaleryloxy-tetradeca-4,6,12-triene-8,10- diyne-1,14-diol	*A. lancea*	300.17	C_19_H_24_O_3_	[83]
235	Erythro-(3*Z*,11*E*)-tridecatriene-7,9-diyne-5,6-diyl-diacetate	*A. lancea*	286.12	C_17_H_18_O_4_	[84]
236	Erythro-(1,5*E*,11*E*)-tridecatriene-7,9-diyne-3,4-diacetate	*A. lancea*	286.12	C_17_H_18_O_4_	[84]
237	Threo-(1,5*E*,11*E*)-Tridecatriene-7,9-diyne-3,4-diacetate	*A. lancea*	286.12	C_17_H_18_O_4_	[84]
238	(3*Z*,5*E*,11*E*)-Tridecatriene-7,9-diynyl-1-*O*-(*E*)-ferulate	*A. lancea*	362.15	C_23_H_22_O_4_	[77]
239	(3*E*,5*E*,11*E*)-Tridecatriene-7,9-diyne-l,2-diacetate	*A. lancea*	286.12	C_17_H_18_O_4_	[84]
240	(3*Z*,5*E*,11*E*)-Tridecatriene-7,9-diyne-1,2-diacetate	*A. lancea*	286.12	C_17_H_18_O_4_	[84]
241	(3*E*,5*Z*,11*E*)-Tridecatriene-7,9-diyne-1,2-diacetate	*A. lancea*	286.12	C_17_H_18_O_4_	[84]
242	(1,5*E*,11*E*)-Tridecatriene-7,9-diyne-3,4-diacetate	*A. chinensis*	226.12	C_17_H_16_O_4_	[61]
243	(4*E*,6*E*,12*E*)-Aetradecatrien-8,10-diyn-1-ol	*A. lancea*	200.12	C_14_H_16_O	[75]
244	(6*E*,12*E*)-Tetradecadiene-triene-8,10-diol	*A. lancea*	300.14	C_18_H_20_O_4_	[75]
245	(10*R*)-Atraethioenyneside A	*A. lancea*	414.13	C_19_H_26_O_8_S	[5]
246	(10*R*)-Atraethioenyneside B	*A. lancea*	386.10	C_17_H_22_O_8_S	[5]
247	(8*S*,9*R*)−2*E*,10*Z*,12-Tridecadiene-4,6-diyne-1,8,9-triol-8-*O*-*β*-D-glucopyranoside	*A. lancea*	380.15	C_19_H_24_O_8_	[5]
248	(8*S*,9*R*)−2*E*,10*Z*,12-Tridecadiene-4,6-diyne-1,8,9-triol-8-*O*-*β*-D-apiofuranosyl-(1 → 6)-*β*-D-glucopyranoside	*A. lancea*	510.21	C_25_H_34_O_11_	[5]
249	(8*S*,9*R*)−2*E*,10*Z*,12-Tridecadiene-4,6-diyne-1,8,9-triol-9-*O*-*β*-D-glucopyranoside	*A. lancea*	380.15	C_19_H_24_O_8_	[5]
250	(8*S*,9*R*)−2*E*,10*Z*,12-Tridecadiene-4,6-diyne-1,8,9-triol-9-*O*-*β*-D-apiofuranosyl-(1 → 6)-*β*-D-glucopyranoside	*A. lancea*	512.19	C_24_H_32_O_12_	[5]
251	(10*S*,11*R*)−2*R*,8*E*,12-Tridecatriene-4,6-diyne-1,10,11- triol-10-*O*-*β*-D-glucopyranoside	*A. lancea*	380.15	C_19_H_24_O_8_	[5]
252	(10*R*,11*R*)−2*E*,8*E*,12-Tridecatriene-4,6-diyne-1,10,11-triol-1-*O*-*β*-D-glucopyranoside	*A. lancea*	380.15	C_19_H_24_O_8_	[5]
253	(10*R*,11*R*)−2*E*,8*E*,12-Tridecatriene-4,6-diyne-1,10,11-triol-1,10-*O*-di-*β*-D-glucopyranoside	*A. lancea*	542.20	C_25_H_34_O_13_	[5]
254	(10*R*,11*R*)−2*E*,8*E*,12-Tridecatriene-4,6-diyne-1,10,11-triol-10-*O*-*β*-D-apiofuranosyl-(1 → 6)-*β*-D-glucopyranoside	*A. lancea*	512.19	C_24_H_32_O_12_	[5]
255	(10*R*,11*S*)−2*R*,8*E*,12-Tridecatriene-4,6-diyne-1,10,11-triol-10-*O*-*β*-D-glucopyranoside	*A. lancea*	380.15	C_19_H_24_O_8_	[5]
256	(10*R*,11*S*)−2*E*,8*E*,12-Tridecatriene-4,6-diyne-1,10,11-triol-10-*O*-*β*-D-apiofuranosyl-(1 → 6)-*β*-D-glucopyranoside	*A. lancea*	512.19	C_24_H_32_O_12_	[5]
257	(10*R*,11*R*)−2*E*,8*E*,12-Tridecatriene-4,6-diyne-1,10,11-triol-10-*O*-*β*-D-glucopyranoside	*A. lancea*	380.15	C_19_H_24_O_8_	[5]
258	(2*E*,8*E*,10*E*,12*R*)-Tridecatriene-4,6-diyne-1,12,13-triol-1,12-di-*O*-*β*-D-glucopyranoside	*A. lancea*	542.20	C_25_H_34_O_13_	[5]
259	(2*E*,8*E*,10*E*,12*R*)-Tridecatriene-4,6-diyne-1,12,13-triol-1-*O*-*β*-D-apiofuranosyl-(1 → 6)-*β*-D-glucopyranoside	*A. lancea*	512.19	C_24_H_32_O_12_	[5]
260	(8*E*,10*E*)-Tetradecadiene-4,6-diyne-3,12,14-triol-3-*O*-*β*-D-glucopyranoside	*A. lancea*	380.15	C_19_H_24_O_8_	[5]
261	(2*E*,8*E*,10*E*,12*R*)-Tetradeca-2,8,10-triene-4,6-diyne-1,2,14-triol-1-*O*-*β*-D-apiofuranosyl-(1 → 6)-*β*-D-glucopyranoside	*A. lancea*	526.21	C_25_H_34_O_12_	[1]
262	(2*E*,8*E*,10*E*,12*R*)-Tetradeca-2,8,10-triene-4,6-diyne-1,12,14-triol-1-*O*-*β*-D-glucopyranoside	*A. lancea*	394.16	C_20_H_26_O_8_	[1]
263	1-(2-furyl)-(7*E*)-Nonene-3,5-diyne-1,2-diacetate	*A. lancea*	300.10	C_17_H_16_O_5_	[84]
264	(2*E*,8*R*)-Decane-4,6-diyne-1,8-diol-8-*O*-*β*-D-glucopyranoside	*A. lancea*	326.14	C_16_H_22_O_7_	[51]
265	(2*E*,8*S*)-Decane-4,6-diyne-1,8-diol-8-*O*-*β*-D-glucopyranoside	*A. lancea*	326.14	C_16_H_22_O_7_	[51]
266	(2*E*,8*R*)-Decene-4,6-diyne-1,8-diol-1-*O*-*β*-D-apiofuranosyl-(1 → 6)-*β*-D-glucopyranoside	*A. lancea*	458.18	C_21_H_30_O_11_	[5]
267	(2*E*,8*R*)-Decane-4,6-diyne-1,8-diol-*O*-di-*β*-D-glucopyranoside	*A. lancea*	488.19	C_22_H_32_O_12_	[51]
268	(*E*)-Deca-2-ene-4,6-diyne-1,10-diol-1-*O*-*β*-D-glucopyranoside	*A. lancea*	326.14	C_16_H_22_O_7_	[40]
269	(*E*)-Deca-2-ene-4,6-diyne-1,10-dio1-1-*O*-*β*-D-apiofuranosyl-(1 → 6)-*β*-D-glucopyranoside	*A. lancea*	458.18	C_21_H_30_O_11_	[40]
270	(2*E*,8*E*,10*R*)-Tridecane-2,8-diene-4,6-diyne-1,10,13-triol-1,13-di-*O*-*β*-D-xylopyranoside	*A. lancea*	544.22	C_25_H_36_O_13_	[5]
271	(2*E*,10*S*)-Tridecatriene-4,6-diyne-1,12,13-triol-13-*O*-*β*-D-glucopyranoside	*A. lancea*	384.18	C_19_H_28_O_8_	[5]
272	(2*E*,10*S*)-Tridecatriene-4,6-diyne-1,12,13-triol-10-*O*-*β*-D-glucopyranoside	*A. lancea*	384.18	C_19_H_28_O_8_	[5]
273	(8*R*,9*R*)−8,9-Dihydroxylatractylodinol-8-*O*-*β*-D-glucopyranoside	*A. lancea*	408.14	C_20_H_24_O_9_	[5]
274	(8*R*,9*R*)−8,9-Dihydroxylatractylodinol-9-*O*-*β*-D-glucopyranoside	*A. lancea*	408.14	C_20_H_24_O_9_	[5]
275	(2*E*,10*S*)-Tetradecadiene-4,6-diyne-1,10,14-triol-10-*O*-*β*-D-apiofuranosyl-(1 → 6)-*β*-D-glucopyranoside	*A. lancea*	530.24	C_25_H_38_O_12_	[5]
276	Atracthioenyneside A	*A. lancea*	414.13	C_19_H_26_O_8_S	[53]
277	Atracthioenyneside B	*A. lancea*	386.10	C_17_H_22_O_8_S	[53]
278	(10*S*,11*R*)-Atracthioenyneside C	*A. lancea*	412.12	C_19_H_24_O_8_S	[5]
279	(10*S*,11*R*)-Atracthioenyneside D	*A. lancea*	412.12	C_19_H_24_O_8_S	[5]
280	(10*R*,11*S*)-Atracthioenyneside E	*A. lancea*	412.12	C_19_H_24_O_8_S	[5]
281	(8*S*)-Decane-4,6-diyne-1,8-diol-*O*-*β*-D-glucopyranoside	*A. lancea*	328.15	C_16_H_22_O_7_	[5]
282	Atractylodemaynes A	*A. macrocephala*	298.16	C_19_H_22_O_3_	[54]
283	Atractylodemaynes B	*A. macrocephala*	314.19	C_20_H_26_O_3_	[54]
284	Atractylodemaynes C	*A. macrocephala*	398.17	C_23_H_26_O_6_	[54]
285	Atractylodemaynes D	*A. macrocephala*	400.19	C_23_H_28_O_6_	[54]
286	Atractylodemaynes E	*A. macrocephala*	412.19	C_24_H_28_O_6_	[54]
287	Atractylodemaynes F	*A. macrocephala*	356.16	C_21_H_24_O_5_	[54]
288	(5*Z*,12*E*)−14-Hydroxytetradeca-5,12-dien-8,10-diyn-1-yl 3-methylbut-2-enoate	*A. macrocephala*	300.17	C_19_H_24_O_3_	[85]
289	5*S*,1'*R*-(6*E*,12*E*)−1-Hydroxytetradeca-6,12-dien-8,10-diyn-5-yl 2-methylbutanoate	*A. macrocephala*	302.19	C_19_H_26_O_3_	[85]
290	(2*E*,8*E*)−12*β*-Methylbutyryltetradeca-2,8-diene-4,6-diyne-1,14-diol	*A. macrocephala*	302.19	C_19_H_26_O_3_	[85]
291	(*E*)−5-[5-(but-3-en-1-yn-1-yl)-thiophen-2-yl]Pent-2-en-4-yn-1-yl acetate	*A. macrocephala*	256.06	C_15_H_12_O_2_S	[85]
292	Erythro-(2*E*,8*E*)−2,8,12-trien-4,6-diyne-10,11-diol	*A. chinensis*	202.10	C_13_H_14_O_2_	[61]
293	(2*E*,8*E*,10*E*)−14-Acetoxy-12-*β*-methylbutyryltetradeca-2,8,10-trien-4,6-diyne	*A. chinensis*	342.18	C_21_H_26_O_4_	[61]
294	(2*E*,8*E*,10*Z*)−13-Acetoxytrideca-2,8,10-trien-4,6-diyne-12-ol	*A. chinensis*	244.11	C_15_H_16_O_3_	[61]
295	(2*E*,8*E*,10*Z*)−13-*β*-Methylbutyryltrideca-2,8,10-trien-4,6-diyne-12-ol	*A. chinensis*	286.16	C_18_H_22_O_3_	[61]
296	(3*Z*,5*E*,11*E*)-Tridecatriene-2-hydroxy-7,9-dinyl-1-*O*-(*E*)-ferulate	*A. lancea*	378.15	C_23_H_22_O_5_	[43]
297	(3*Z*,5*E*,11*E*)-Tridecatriene-2-acetoxy-7,9-dinyl-1-*O*-(*E*)-ferulate	*A. lancea*	420.16	C_25_H_24_O_6_	[43]
298	(3*E*,5*Z*,11*E*)-Tridecatriene-2-acetoxy-7,9-dinyl-1-*O*-(*E*)-ferulate	*A. lancea*	420.16	C_25_H_24_O_6_	[43]
299	(3*E*,5*E*,11*E*)-Tridecatriene-2-ace-toxy-7,9-dinyl-1-*O*-(*E*)-ferulate	*A. lancea*	434.17	C_26_H_26_O_6_	[43]
300	(5*E*,11*E*)−3*S*-Ace-toxy-4*S*-*O*-(3-methylbutanoyl)−1,5,11-tridecatriene-7,9-diyne-3,4,13-triol	*A. lancea*	358.18	C_21_H_26_O_5_	[43]
301	(6*E*,12*E*)−5*S*-*O*-(3-methylbutanoyl)−6,12-Didecadiene-8,10-diyne-1,5-diol	*A. lancea*	316.20	C_20_H_28_O_3_	[43]
302	1-*O*-Feruloyltetradeca-4*E*,6*E*,12*E*-trien-8,10-diyne	*A. lancea*	390.18	C_25_H_26_O_4_	[43]
303	(3*Z*,5*E*,11*E*)-Tridecatriene-7,9-diynyl-1-*O*-(*E*)-ferulate	*A. lancea*	362.15	C_23_H_22_O_4_	[43]
304	(2*E*,8*E*)−12-*β-*Methylbutyryltetradeca-2,8,10-trien-4,6-diyne-1,14-diol	*A. lancea*	434.21	C_27_H_30_O_5_	[42]
305	(2*E*,8*E*,10*E*)−12-Acetoxy-14-feruloyltetradeca-2,8,10-trien-4,6-diyne-1-ol	*A. lancea*	300.17	C_19_H_24_O_3_	[42]
306	(2*E*,8*E*)−1-Hydroxynona-2,8-dien-4,6-diyne-1'-methoxyfuran-4'-one	*A. lancea*	244.07	C_14_H_12_O_4_	[42]
307	Erythro-(2*E*,10*Z*)−1,8,9-triacetoxytrideca-2,10,12-triene-4,6-diyne-1,14-diol	*A. lancea*	344.13	C_19_H_20_O_6_	[42]
308	Atracetylenes A	*A. macrocephala*	420.19	C_26_H_28_O_5_	[86]
309	Atracetylenes B	*A. macrocephala*	434.17	C_26_H_26_O_6_	[86]
310	Atracetylenes C	*A. macrocephala*	434.17	C_26_H_26_O_6_	[86]
311	Atracetylenes D	*A. macrocephala*	492.21	C_29_H_32_O_7_	[86]
312	Atracetylenes E	*A. macrocephala*	492.21	C_29_H_32_O_7_	[86]
313	Atracetylenes F	*A. macrocephala*	290.15	C_17_H_22_O_4_	[86]
314	2,8-Dimethyl-6-hydroxy-2-(4-methyl-3-pentenyl)−2H-chromene	*A. lancea*	258.16	C_17_H_22_O_2_	[68]
315	2-[(2*E*)−3,7-dimethyl-2,6-octadienyl]−6-Methyl-2,5- cyclohexadiene-1,4-dione	*A. lancea*	258.16	C_17_H_22_O_2_	[68]
316	2-[(2′*E*)−3′,7′-dimethyl-2′,6′-octadienyl]−4-Methoxy-6- methylphenol	*A. lancea*	274.19	C_18_H_26_O_2_	[77]
317	4-Hydroxy-3-methoxyphenol-*β*-D-glucopyranoside	*A. lancea*	302.10	C_13_H_18_O_8_	[55]
318	Seguinoside B	*A. lancea*	434.14	C_18_H_26_O_12_	[55]
319	Syringin	*A. lancea*	372.14	C_17_H_24_O_9_	[55]
320	4-Hydroxy-3-methoxyphenol-*β*-D-apiopyranosyl-(1 → 6)-D-glucopyranoside	*A. lancea*	434.14	C_18_H_26_O_12_	[55]
321	4-Hydroxy-3-methoxyphenyl-*β*-xylopyranosyl-(1 → 6)-*β*-glucopyranoside	*A. lancea*	434.14	C_18_H_26_O_12_	[55]
322	Icariside F2	*A. lancea*	402.39	C_18_H_26_O_10_	[55]
323	Adenosine	*A. lancea*	267.10	C_10_H_13_N_5_O_4_	[55]
324	Phenethyl-*α*-L-rhamnopyranosyl-(1 → 6)-*β*-D-glucopyranoside	*A. lancea*	402.15	C_18_H_26_O_10_	[39]
325	Scopoletin-D-xylopyranosyl-(1 → 6)-D-glucopyranoside	*A. lancea*	486.14	C_21_H_26_O_12_	[39]
326	1,3-di-*O*-Caffeoylquinic acid	*A. lancea*	516.13	C_25_H_24_O_12_	[53]
327	Chlorogenic acid	*A. lancea*	354.10	C_16_H_18_O_9_	[53]
328	5-*O*-Feruloylquinic acid	*A. lancea*	354.10	C_16_H_18_O_9_	[53]
329	Osthol	*A. lancea*	244.11	C_15_H_16_O_3_	[68]
330	5-Hydroxymethyl furaldehyde	*A. lancea*	126.03	C_6_H_6_O_3_	[75]
331	L-Phenylalanine	*A. lancea*	165.08	C_9_H_11_NO_2_	[55]
332	L-Tryptophan	*A. lancea*	204.09	C_11_H_12_N_2_O_2_	[55]
333	*trans*−2-Hydroxyisoxypropyl-3-hydroxy-7-isopentene-2,3-dihydrobenzofuran-5-carboxylic acid	*A. lancea*	306.15	C_17_H_22_O_5_	[46]
334	Uridine	*A. lancea*	244.07	C_9_H_12_N_2_O_6_	[45]
335	Thymidine	*A. lancea*	242.09	C_10_H_14_N_2_O_5_	[45]
336	(−)-(1*S*,3*S*)−1-Methyl-1,2,3, 4-tetrahydro-*β*-carboline-3-carboxylic acid	*A. lancea*	230.11	C_13_H_14_N_2_O_2_	[45]
337	Adenine	*A. lancea*	135.05	C_5_H_5_N_5_	[45]
338	(7*R*, 8*S*)−4,7,9,3',9'-Pentahydroxy-3-methoxyl-8-4'-oxy-neolignan-3'-*O*-*β*-D-glucopyranoside	*A. lancea*	526.21	C_25_H_34_O_12_	[73]
339	(7*S*, 8*R*)−4,7,9,3',9'-Pentahydroxy-3-methoxyl-8-4'-oxy-neolignan-3'-*O*-*β*-D-glucopyranoside	*A. lancea*	526.21	C_25_H_34_O_12_	[73]
340	Syringaresinol-4'-*O*-*β*-D-glucopyranoside	*A. lancea*	580.22	C_28_H_36_O_13_	[73]
341	Secoisolariciresin-4-yl-*O*-*β*-D-glucopyranoside	*A. lancea*	524.23	C_26_H_36_O_11_	[73]
342	(*E*)-Isoconiferin	*A. lancea*	372.14	C_17_H_24_O_9_	[39]
343	(7*E*)-Sinapate-4-*O*-*β*-D-glucopyranoside	*A. lancea*	386.12	C_17_H_22_O_10_	[39]
344	Cichoriin	*A. lancea*	340.08	C_15_H_16_O_9_	[39]
345	Scopoletin-7-*O*-*α*-L-rhamnopyranosyl-(1 → 6)-*β*-D-glucopyranoside	*A. lancea*	486.14	C_21_H_26_O_13_	[39]
346	Isoscopoletin-6-*O*-*β*-D-glucopyranoside	*A. lancea*	354.10	C_16_H_18_O_9_	[39]
347	(1*S*,3*S*)−1-Methyl-1,2,3,4-tetrahydro-*β*-carboline-3-carboxylic acid	*A. lancea*	230.11	C_13_H_14_N_2_O_2_	[39]
348	p-Hydroxybenzoic acid-4-*O*-*β*-D-glucopyranosyl-(1 → 3)-*α*-L-rhamnopyranoside	*A. lancea*	446.14	C_19_H_26_O_12_	[56]
349	Vanillic acid-4-*O*-*β*-D-glucopyranosyl-(1 → 3)-*α*-L-rhamnopyranoside	*A. lancea*	476.15	C_20_H_28_O_13_	[56]
350	**3**	*A. lancea*	322.14	C_17_H_22_O_6_	[56]
351	1''-Hydroxylcrotonine	*A. lancea*	266.09	C_12_H_14_N_2_O_5_	[56]
352	Crotonine	*A. lancea*	250.10	C_12_H_14_N_2_O_4_	[56]
353	**5**	*A. lancea*	596.21	C_28_H_36_O_14_	[56]
354	**6**	*A. lancea*	596.21	C_28_H_36_O_14_	[56]
355	Longi-floroside B	*A. lancea*	568.22	C_27_H_36_O_13_	[56]
356	Secoisolariciresinol-9-*O*-*β*-D-glucopyranoside	*A. lancea*	524.23	C_26_H_36_O_11_	[56]
357	Puerarin	*A. lancea*	416.11	C_21_H_20_O_9_	[56]
358	3'-Methoxy puerarin	*A. lancea*	446.12	C_22_H_22_O_10_	[56]
359	7-Methylatractylochromene	*A. chinensis*	272.18	C_18_H_24_O_2_	[61]
360	(+)-Atralignanine A	*A. macrocephala*	330.15	C_19_H_22_O_5_	[58]
361	Atralignanine A	*A. macrocephala*	330.15	C_19_H_22_O_5_	[58]
362	(+)-Atralignanine B	*A. macrocephala*	372.12	C_20_H_20_O_7_	[58]
363	Atralignanine B	*A. macrocephala*	372.12	C_20_H_20_O_7_	[58]
364	Atramollugin A	*A. macrocephala*	346.14	C_19_H_22_O_6_	[58]
365	Atramindanone A	*A. macrocephala*	176.08	C_11_H_12_O_2_	[58]
366	2,6,3',6'-Tetra (3-methylbutanoyl) sucrose	*A. lancea*	676.37	C_33_H_56_O_14_	[57]
367	2,4,3',6'-Tetra (3-methylbutanoyl) sucrose	*A. lancea*	676.37	C_33_H_56_O_14_	[57]
368	2,6,3',4'-Tetra (3-methylbutanoyl) sucrose	*A. lancea*	592.31	C_28_H_48_O_13_	[57]
369	2,4,3',4'-Tetra (3-methylbutanoyl) sucrose	*A. lancea*	592.31	C_28_H_48_O_13_	[57]
370	2,1',3',6'-Tetra (3-methylbutanoyl) sucrose	*A. lancea*	662.39	C_33_H_58_O_13_	[57]
371	3',4',6'-Tris (3-methylbutanoyl)−1’-(2-methylbutanoyl) sucrose	*A. lancea*	578.33	C_28_H_50_O_12_	[57]

**Table 2:** Anti-cancer effects

Clinical application	Extracts/Compounds	Models	Mechanisms of action	References
Cholangiocarcinoma	*A. lancea* ethanol extract	CL-6 cells		[197]
	*A. lancea* ethanol extract/*β*-eudesmol	CCA-xenograft nude mouse model		[89]
	*A. lancea* ethanol extract	*Opisthorchis viverrini*/dimethylnitrosamine-induced CCA hamster model		[90]
	atractylodin/*β*-eudesmol	CL-6 and HUCCT-1 cells	Induce G1 phase cell cycle arrest, activate caspase-3/7-mediated apoptosis	[6]
		CL-6 cells	Suppress multiple key signaling pathways such as HO-1, STAT1/3, and NF-κB	[94, 95]
	*β*-eudesmol	Human CCA KKU-100 cells	Suppression of NQO1 protein and activation of Bax/Bcl-2 protein expression ratio	[96]
	Atractylodin	HUCCT-1 cells	PI3K/AKT/mTOR and p38MAPK	[97]
Gastric cancer	PE fraction of *A. lancea* extract	BGC-823 and SGC-7901 cells		[98]
	AT-I	MGC-803 cells	Notch	[99]
	AT-II	HGC-27 and AGS Cells	Akt/ERK	[100]
	AT-III	Gastric precancerous lesions rats	Decrease of angiogenesis-associated HIF-1α and VEGF-A, and downregulation of DLL4	[101]
Lung cancer	AT-I	A549 and HCC827 cells A549-Luc-xenograft nude mouse model	PDK1	[102]
	AT-II	A549 cells Lewis lung carcinoma mouse xenograft and metastasis models	Inhibits M2-like polarization	[103]
Liver cancer	AT-II	Hep3B and Huh7 cells BALB/c nude mice	TRAF6/NF-κB	[104]
	AT-III	HepG2 and SMMC7721 cells	miR-195-5p/FGFR1	[105]
	Atractylon	HepG2, SMCC7721, and MHCC97H cells	Inhibits EMT	[106]
	Atractylodin	Huh7 and Hccm cells	Inhibits the expression of GPX4 and FTL proteins, and up-regulats the expression of ACSL4 and TFR1 proteins	[107]
Breast cancer	AT-I	MCF-7 and MDA-MB-231 cells N-Nitroso-N-methylurea induced rat breast cancer models	TLR4-mediated NF-κB	[109]
	AT-II	MCF 10 A cells N-Nitroso-N-methylurea-induced rat	Nrf2-ARE	[108]
	Atractylodin	MCF-7 cells	P13K/Akt/mTOR	[110]
Triple-negative breast cancer	*A. lancea* crude extract	MDA-MB-231 cells	Modulate EMT markers, reduce cell motility, and inhibiting NF-κB signaling	[111]
	AT-I	MDA-MB-231 cells	Blocks CTGF expression and fibroblast activation	[113]
	AT-I	MDA-MB-231 cells	Downregulates the expression of *TPI1* and *GPI*	[112]
Castration-resistant prostate cancer	AT-I	AR-positive C4-2 and AR-negative PC-3 cells C4-2B-EnzR cells-xenograft nude mouse model	KIF15	[114]
	AT-I			
Clear cell renal cell carcinoma	AT-I	HK-2, ACHN, 786O, and OSRC2 cells 786O cells-xenograft nude female athymic mouse model	ATP6V0D2-mediated autophagic degradation of EPAS1/HIF2α	[115]
Endometrial cancer	AT-II	RL95-2 and AN3CA cells	PADI3-ERK	[116]
Glioblastoma	AT-II	U-87 and U-251 cells	ERK/MAPK	[117]
Colorectal cancer	AT-II	HT29 and HCT15 cells Lung metastatic C57BL/6 mice	NF-κB p65/PD-L1	[118]
Cervical cancer	AT-III	HeLa and SiHa cells	Regulates IGF2BP3 through ETV5	[119]

**Table 3:** Anti-inflammatory effects

Clinical application	Extracts/Compounds	Models	Mechanisms of action	References
Gastroenteritis	*A. chinensis* ethanol extract	HCl/EtOH-induced gastritis rat model	Akt/IκBα/NF-κB	[120]
	Atractylodin	Constipation-prominent and diarrhea-prominent rats	NF-κB	[7]
	AT-I	DSS-induced UC mouse model	SPHK1/PI3K/AKT	[121]
		DSS-induced UC mouse model	S100A9/AMPK/mTOR	[122]
	AT-III	TNBS-induced mouse colitis model	FPR1/Nrf2	[123]
		DSS-induced mice and LPS- stimulated IEC-6	AMPK/SIRT1/PGC-1α	[124]
	Hinesol	DSS-induced UC mouse model	Suppresses Src-mediated NF-κB and chemokine	[125]
Vascular inflammation	AT-I	FCA induced mouse air pouch model as well as the mice aortic ring co-cultured with peritoneal macrophages model	Downregulates the production of NO and the expression of TNF-α, IL-1β, IL-6, VEGF, and PlGF	[126]
	AT-I	Ox-LDL-induced VSMCs and macrophage models	Restores HO-1 expression and inhibits the p38 MAPK/NF-κB	[127]
Neuroinflammation	ALR-ELNs	BV-2 murine microglial cells	Downregulate TLR4 expression	[128]
	AT-III	In vivo MCAO mouse model and an in vitro OGDR model in primary microglial cells	JAK2/STAT3/Drp1	[129]
	AT-III	LPS-stimulated mouse microglial cell line MG6	Decreases the mRNA expression and protein levels of TLR4	[130]
	AT-III	A rat spinal cord injury model and LPS-stimulated BV2 microglial line	Promotes microglial/macrophage polarization from M1 to M2	[25]
Osteoarthritis	AT-III	A rat osteoarthritis (OA) model, human OA cartilage explants, and both rat and human primary chondrocyte culture models	NF-κB	[131]
Asthma	AT-III	IL-4-induced human bronchial epithelial cells and ovalbumin-induced asthmatic mouse model	Reduces NLRP3 inflammasome activation and Th1/Th2 imbalances	[132]

**Table 4:** Neuroprotective effects

Clinical application	Extracts/Compounds	Models	Mechanisms of action	References
Cognitive improvement	*A. macrocephala* aqueous extract	Focal cerebral ischemia/reperfusion rat model	Increases activities of the antioxidants SOD, GSH, and CAT	[8]
	*A. macrocephala* aqueous extract	D-galactose-induced brain aging mouse model	Increases expression of Syn, PKC, and CREB proteins in the hippocampus	[133]
	Biatractylenolide	D-galactose-induced brain aging mouse model	Reduces ROS formation, decreases AChE activity, and upregulates synaptic-related protein expression	[134]
	AT-III	Isoflurane-induced neuronal injury rat model	PI3K/Akt/mTOR	[135]
	AT-III	Intracerebroventricular-streptozotocin induced Alzheimer's disease rat model	PI3K/AKT/GSK3β	[136]
	AT-III	Chronic high-dose homocysteine-induced cognitive impairment rat model	Reduces ROS formation, restores p-PKC expression	[137]
	Atractylon	CIH-exposed mice and CIH-induced BV2 cells	Upregulation of SIRT3 expression	[138]
Glutamate-induced excitotoxicity	Biatractylolide	Glutamate-induced cell damage in PC12 and SH-SY5Y cells	PI3K-Akt-GSK3β	[139]
	AT-I	MPP-induced human dopaminergic SH-SY5Y cell model	Suppresses the mitochondrial apoptotic pathway and enhances the antioxidant defense through upregulation of OH-1	[140]
	AT-III	Cerebral cortical neurons from BALB/c mouse embryos	Suppression of caspase signaling	[141]
Alzheimer′s disease	Biatractylolide-II	In vitro model of AChE from electric eels	Inhibits AChE	[142]
	*A. macrocephala* ethanol extract	Alzheimer's disease transgenic *C. elegans* CL2241 model	LKB1-AMPK-TFEB	[143]
	Biatractylolide	Amyloid-β-induced memory impairment in a rat model	NF-κB	[144]
	AT derivative	*C. elegans* CL4176, HT22 cells, and APP/PS1 mouse models	Exhibits potent LSD1 inhibition,	[145]
	AT-III	*C. elegans* CL4176, SH-SY5Y APP_SWE_, and APP/PS1 mouse models	YY1-TFEB	[146]
Parkinson's disease	AT-I	LPS-stimulated BV-2 microglial cells and MPTP-induced C57BL/6J mouse model of Parkinson's disease	NF-κB	[147]
Anti-depression and anti-anxiety	AT-I	CUMS-induced mouse model of depression	Inhibits NLRP3 inflammasome activation and reduces IL-1β production	[148]
	AT-III	Corticosterone-induced PC12 cells	Mitochondrial apoptotic and MAPKs/NF-κB	[149]
	Atractylodin	CUMS-induced mouse model of depression	Regulates ferroptosis and synaptic plasticity	[150]

**Table 5:** Gastrointestinal function-improving effects

Clinical application	Extracts/Compounds	Models	Mechanisms of action	References
Gastrointestinal motility regulation	*A. lancea* extract/*β*-eudesmol	Atropine-, dopamine-, and 5-HT-treated mouse models of gastrointestinal dysmotility	Inhibits the dopamine D_2_ receptor and 5-HT_3_ receptor	[151]
	*A. macrocephala* ethanol extract	Neostigmine-induced hypermotility mouse model	Inhibits the pacemaker activity of interstitial cells of Cajal and modulates the cAMP/ATP-sensitive K⁺ channel pathway	[9]
Intestinal dysfunction	Bran-fried *A. macrocephala* aqueous extract	Spleen deficiency diarrhea pregnant mouse model	Modulates intestinal barrier and water-fluid metabolism	[152]
	AT-III	Pyrotinib-induced Wistar rat diarrhea model	AMPK/CFTR	[153]
	*A. macrocephala* aqueous extract	The senna leaf extract-induced rat model of spleen-deficiency constipation	Upregulation of the TGR5 receptor	[154]
	*A. macrocephala* aqueous extract	Loperamide-induced slow transit constipation rat model	Upregulation of TPH and downregulation of MAO and IDO key enzyme expressions	[155]
Gastric mucosal protection and ulcer	Bran-processed *A. lancea*	Acetic acid-induced gastric ulcer rat model	Upregulates EGF and TFF2 while downregulates TNF-α, IL-8, IL-6, and PGE2	[156]
	Atractyloside A	Spleen-deficiency syndrome rat model	p38 MAPK	[157]
	AT-I	Indomethacin-induced gastric ulcer rat model	Inhibits NLRP3 inflammasome activation and increases prostaglandin E₂ levels	[158]
	AT-III	Ethanol-induced primary rat gastric mucosal cell damage and acute gastric ulcer rat model	Suppression of MMP-2 and MMP-9	[159]
	AT-I	Multiple *H. pylori* strains	Direct inhibition of *H. pylori* growth (via a non-urease-dependent pathway)	[160]
Irritable bowel syndrome	*A. macrocephala* ethanol extract	Zymosan-induced irritable bowel syndrome mouse model	Modulates TRPV1, NaV1.5, and NaV1.7 channels	[161]
	AT-I	H₂O₂-treated NCM460 cells	Modulates the miR-34a-5p-LDHA signaling pathway and restoring glucose metabolism	[162]
	AT-I	IEC-6 cells	Polyamine-mediated Ca^2+^ signaling pathway	[163]
Dysbiosis of the intestinal microbiome	AT-I	Dysbacteriosis mouse model	TLR4/MyD88/NF-κB	[164]

**Table 6:** Hepatoprotective effects

Clinical application	Extracts/Compounds	Models	Mechanisms of action	References
Liver fibrosis	AT-III	CCl₄-induced and bile duct ligation mouse models of liver fibrosis along with human hepatic stellate cells line LX-2 and rat HSC line T6	Inhibits ASCT2, and regulates glutaminolysis-associated IL-1α/NF-κB feedback loop	[10]
	AT-III	Hepatic stellate cells	cGAS/NF-κB	[165]
	AT-III	Bile duct ligation mouse model and TGF-β1-activated LX-2 cells	Inhibits the PI3K/AKT pathway and regulates glutamine metabolism	[166]
Non-alcoholic fatty liver disease	AT-III	High-fat diet-induced NAFLD mouse model	Activates hepatic AdipoR1-mediated AMPK/SIRT1 signaling pathway	[167]
	Atractylodin	High-fat diet-induced NAFLD mouse model	Regulates Nrf2-mediated ferroptosis	[168]
Drug-induced liver injury	AT-I	APAP-induced liver injury mouse model	TLR4/MAPKs/NF-κB	[169]
Acute liver failure	AT-I	LPS/D-GalN-induced acute liver failure mouse model	Regulates M1 macrophage polarization	[170]

**Table 7:** Lung protection effects

Clinical application	Extracts/Compounds	Models	Mechanisms of action	References
Pulmonary fibrosis	AT-III	Bleomycin-induced experimental pulmonary fibrosis and oxidative stress mouse model	Nrf2/NQO1/HO-1	[171]
	Atractylodinol	TGF-β1-induced A549 and HFL1 cells and a bleomycin-induced pulmonary fibrosis in C57BL/6J mice	Inhibits TGF-β receptor 1 recycling by stabilizing vimentin	[11]
Pulmonary injury	*A. japonica* ethanol extract	PM₂.₅-induced subacute lung injury mouse model	p38 MAPKα and PI3K/Akt	[172]
	*A. japonica* ethanol extract	PM₂.₅-induced subacute lung injury mouse model	Downregulates mucus-related genes, increases the levels of acetylcholine and substance P, and inhibits the expression of inflammatory protein COX-2 and apoptotic protein cleaved caspase-3	[173]
	AT-I	LPS-induced acute lung injury mouse model	TLR4/NF-κB	[174]
	AT-III	Sepsis-mediated lung injury mouse model	Inhibition of FoxO1 and VNN1 protein	[15]

**Table 8:** Other effects

Clinical application	Extracts/Compounds	Models	Mechanisms of action	References
Skeletal muscle atrophy	*A. macrocephala* aqueous extract	Dexamethasone-induced skeletal muscle atrophy mouse model	Improving mitochondrial function	[175]
Osteoporosis	AT-I	RANKL-stimulated RAW264.7 cell osteoclast differentiation model and dexamethasone-induced glucocorticoid osteoporosis mouse model	NF-κB	[176]
Cardiac fibrosis	AT-III	Left anterior descending coronary artery permanent ligation-induced myocardial infarction mouse model and a TGF-β1-stimulated mouse cardiac fibroblast model	Suppression of the RhoA/ROCK1 and ERK1/2	[177]
Fetal cardiac developmental abnormalities	AT-I	Streptozotocin-induced diabetes mellitus mouse model	Inhibition of the ROS-activated STAT3	[178]
Insulin resistance	*A. macrocephala*-*Cuscuta chinensis* Lam. ethanol extract	Insulin resistance-induced diabetic fly model	PI3K/Akt	[179]
Inhibit melanin production	ALR-ELNs	α-MSH-stimulated B16-F10 melanoma cell model	Downregulates MITF and reduces expression of melanogenic enzymes, leading to decreased tyrosinase activity	[180]
Ischemic stroke	Combination of AT-I, AT-III, and Paeoniflorin	MCAO-induced mouse models of cerebral ischemia and OGD-treated bEnd.3 cell models	Upregulates IGF-2 and activates the PI3K/AKT and Ras/Raf/ERK pathways	[181]
Acute kidney injury	AT-III	Cisplatin- and ischemia–reperfusion-induced mouse model of acute kidney injury and hypoxia/reoxygenation-treated HK-2 cell model	PI3K/AKT	[182]
Post-traumatic joint contracture	AT-III	Post-traumatic knee fibrosis mouse model and TGF-β1-induced synovial fibroblast model	TGF-β/Smad	[183]

In studies on NAFLD, AT-III exerts hepatoprotective effects by activating the AdipoR1-mediated AMPK/SIRT1 signaling pathway in the liver [167]. Additionally, atractylodin effectively suppresses hepatocyte ferroptosis by upregulating key upstream proteins of the Nrf2 signaling pathway, including GPX4, FTH1, and SLC7A11, thereby ameliorating NAFLD progression [168].

Furthermore, in studies on drug-induced liver injury, AT-I confers protection against APAP-induced hepatotoxicity through mediation of the TLR4/MAPK/NF-κB pathway [169]. In acute liver failure, pretreatment with AT-I attenuates LPS/D-GalN-induced acute liver failure symptoms, partially through modulation of M1 macrophage polarization [170], Table 6

### Lung protection effects

In pulmonary fibrosis research, AT-III has been demonstrated to significantly ameliorate bleomycin-induced pulmonary fibrosis by activating the Nrf2/HO-1 signaling pathway and suppressing oxidative stress [171]. On the other hand, atractylodinol directly binds to vimentin, thereby inhibiting the recycling of TGF-β receptor 1 and its downstream signaling, offering a novel targeting strategy for pulmonary fibrosis treatment [11].

In pulmonary injury studies, the ethanol extract of *A. japonica* effectively alleviates PM2.5-induced lung injury by inhibiting the p38 MAPKα and PI3K/Akt signaling pathways [172], while also exhibiting unique mucolytic activity. Its mechanism is closely associated with significant elevation of pulmonary substance P and acetylcholine levels, demonstrating comparable efficacy to the mucolytic agent ambroxol hydrochloride in improving mucus secretion and respiratory acidosis, without hepatotoxicity risks [173]. Furthermore, AT-I exerts protective effects against LPS-induced acute lung injury through suppression of the TLR4/NF-κB pathway [174], whereas AT-III mitigates sepsis-mediated lung injury by inhibiting FoxO1 and VNN1 proteins [15], Table 7.

### Other effects

Furthermore, plants of the genus *Atractylodes* and their active constituents demonstrate broad pharmacological potential across various pathological conditions Table 8. In the skeletal system, the aqueous extract of *A. macrocephala* alleviates dexamethasone-induced skeletal muscle atrophy in mice by improving mitochondrial function [175]. Additionally, AT-I effectively suppresses RANKL-induced osteoclast formation through inhibition of the NF-κB signaling pathway and protects against glucocorticoid-induced bone loss, suggesting its potential as a therapeutic agent for glucocorticoid-induced osteoporosis [176].

In the cardiovascular and metabolic field, AT-III demonstrates potential in suppressing cardiac fibrosis following myocardial infarction by alleviating oxidative stress, reducing inflammation, and inhibiting cardiac fibroblast activation [177]. AT-I significantly lowers the risk of fetal cardiac developmental abnormalities in a gestational diabetes model through inhibition of the ROS-activated STAT3 pathway. Furthermore, it indirectly protects normal fetal heart development by modulating maternal gut microbiota and suppressing the EGFR/STAT3 pathway, representing the first documented instance of AT-I's effectiveness in this area [178]. On the other hand, the *A. macrocephala*-*Cuscuta chinensis* Lam. ethanol extract effectively improves glucose metabolism in diabetic Drosophila, prevents body weight loss and triglyceride reduction, enhances PI3K/Akt pathway activity and glucose uptake capacity, and also rescued diabetes-induced dFoxO nuclear localization [179].

Furthermore, ALR-ELNs are efficiently taken up by melanoma cells and significantly suppress melanogenesis by downregulating microphthalmia-associated transcription factor expression and subsequently inhibiting the expression of melanin synthesis-related enzymes, demonstrating their potential as a new whitening agent in pharmaceuticals and cosmetics [180]. The combination of AT-I, AT-III, and Paeoniflorin promotes post-ischemic angiogenesis and neurological functional recovery through specific activation of the IGF-2 signaling pathway [181]. AT-III directly binds to and activates PIK3CA, and enhance AKT signaling, thereby exerting superior renoprotective effects compared to curcumin in acute kidney injury [182]. Furthermore, this compound targets Sirt1 and promotes Smad3 deacetylation, consequently inhibiting the proliferation, migration, and collagen deposition of synovial fibroblasts, ultimately alleviating post-traumatic knee joint fibrosis [183].

### Safety and toxicity

TCM formulas containing *Atractylodes* species, primarily *A. macrocephala* and *A. lancea*, have a long-standing history of clinical use and are widely recognized for their safety and therapeutic efficacy [184]. However, with their expanding global utilization, notably their adoption as an alternative strategy to prevent COVID-19 infection among high-risk populations [185, 186], the imperative to thoroughly elucidate their underlying toxicological characteristics remains critically urgent. According to TCM principles, the adverse reactions associated with *Atractylodes* species are predominantly driven by their intense "dryness" property, which readily consumes Yin fluids, thereby leading to the impairment of spleen Yin and subsequent dysfunction of the spleen in transportation. Therefore, their clinical use should be strictly avoided in patients characterized by internal heat coupled with Yin deficiency and profuse sweating [4]. Accumulating evidence indicates that the "dryness" of *Atractylodes* species is closely associated with their volatile oil components. Traditional processing methods, such as bran-frying and rice water-processing, serve as a primary mechanism to alleviate this potent "dryness" and maximize safety by substantially decreasing the concentration of these volatile constituents [187].

Modern toxicological and pharmacological investigations have substantiated the safety profiles of *Atractylodes* extracts across diverse animal models. Specifically, studies showed that following the administration of an *A. lancea* ethanol extract at doses up to 5,000 mg/kg body weight, no significant systemic toxicity was observed in rats and mice, with manifestations restricted to gastric irritation and mild central nervous system depressant signs, such as diminished tactile and balance responses, alongside reduced alertness and locomotor activity [188]. In parallel, *A. macrocephala* has exhibited an equally robust safety profile in rodent assays. Mice administered with an *A. macrocephala* aqueous extract showed no detectable toxic responses or behavioral alterations, even at extraordinarily high doses of up to 100 g/kg via intraperitoneal injection and 20 g/kg through intramuscular injection [189]. Similarly, a 90-day oral toxicity study of its volatile oil emulsion (up to 300 mg/kg) in rats showed no histopathological damage, though male rats exhibited retarded body weight gain [190].

To date, cumulative evidence from several clinical trials indicates that the genus *Atractylodes* exhibits a favorable safety profile when integrated into multi-herb formulations, for instance, Fufang Cangzhu Tang demonstrated excellent tolerability over 8 weeks of continuous administration [191]. Similarly, long-term treatment with AT-I for up to 7 weeks in patients with cancer cachexia presented no severe adverse events [192].

Despite the general safety profiles, emerging evidence highlights potential tissue-specific toxicities and developmental liabilities associated with individual chemical entities. Particularly, atractylodin, a prominent acetylenic compound present in diverse *Atractylodes* extracts, was confirmed to possess embryotoxic effects in the zebrafish model [193]. Most critically, another component, atractyloside, poses severe clinical hazards. Its exposure has been documented to cause fatal renal proximal tubule necrosis and centrilobular hepatic necrosis in both humans and livestock [194].

In addition to individual chemical toxicities, potential drug-drug interactions and metabolic risks pose a significant concern. It has been demonstrated that AT-I enhances coumarin-induced hepatotoxicity. Specifically, in rifampicin-induced HepG2 cells, the ternary combination of coumarin, astragaloside IV, and AT-I provoked severe synergistic cytotoxicity (reducing cell viability to ~ 40%), mimicking high-dose coumarin toxicity and exposing a critical polypharmacy risk [195]. Concurrently, metabolic bottlenecks may arise from enzyme inhibition. AT-I and III possess strong, specific inhibitory activities against UDP-glucuronosyltransferase 2B7, a major Phase II enzyme involved in the metabolism of various clinical medications [196].

## Conclusions and prospects

Although plants of the genus *Atractylodes* serve as indispensable medicinal and edible resources with 371 isolated compounds and diverse pharmacological profiles, transforming them from traditional remedies into modern therapeutics requires overcoming several critical bottlenecks. To address these gaps, future research must evolve from broad bioactivity screening toward precise mechanistic exploration. First, phytochemical profiling should be expanded beyond mainstream species to understudied species such as *A. japonica* and *A. carlinoides*, focusing on identifying rare constituents with novel structural skeletons that could serve as leads for drug discovery. Concurrently, identifying the direct molecular targets of core compounds through chemical biology approaches will help elucidate SAR, thereby facilitating structural optimization and rational drug design. Furthermore, although traditional processing has been widely reported to enhance therapeutic efficacy while mitigating the adverse effects associated with long periods of or excessive consumption, further systematic studies are needed to comprehensively map these chemical transformations and optimize current quality control standards. Given the established clinical efficacy of *Atractylodes* in managing gastrointestinal disorders and its documented regulatory effects on the microbiome, further research is warranted to deeply clarify the continuous metabolic interactions between its constituents and the gut microbiota, particularly exploring how microbial metabolites modulate host signaling pathways to maintain intestinal homeostasis. Ultimately, advancing toward rigorous clinical trials with standardized formulations will establish a framework characterized by clear mechanisms, precise material bases, and verifiable efficacy.

## Data Availability

No datasets were generated or analysed during the current study.

## Abbreviations

5-HT: 5-Hydroxytryptamine
AChE: Acetylcholinesterase
ACSL4: Acyl-CoA synthetase long-chain family member 4
AdipoR1: Adiponectin receptor 1
AKT: Protein kinase B
ALR-ELNs: *A. **lancea* rhizome-derived exosome-like nanoparticle
AMPK: AMP-activated protein
APAP: Acetaminophen
APP: Amyloid precursor protein
ARE: Antioxidant response element
AR-V7: Androgen receptor variant 7
ASCT2: Alanine-serine-cysteine transporter 2
AT-I: Atractylenolide I
AT-II: Atractylenolide II
AT-III: Atractylenolide III
ATP: Adenosine triphosphate
ATP6V0D2: ATPase H^+^ transporting V0 subunit D2
Aβ: Amyloid-β
B4GALT2: Beta-1,4-galactosyltransferase2
cAMP: Cyclic adenosine monophosphate
Caspase: Cysteinyl aspartate specific proteinase
CCA: Cholangiocarcinoma
CFTR: Cystic fibrosis transmembrane conductance regulator
cGAS: Cyclic GMP-AMP synthase
CIH: Chronic intermittent hypoxia
CMC: Chemistry, manufacturing, and controls
CTGF: Connective tissue growth factor
CUMS: Chronic unpredictable mild stress
dFoxO: *Drosophila* Forkhead box O
D-GalN: D-Galactosamine
DLL4: Delta-like ligand 4
Drp1: Dynamin-related protein 1
DSS: Dextran sulfate sodium
EGF: Epidermal growth factor
EMT: Epithelial-mesenchymal transition
EPAS1: Endothelial PAS domain-containing protein 1
ERK: Extracellular signal-regulated kinase
ETV5: ETS translocation variant 5
FGFR1: Fibroblast growth factor receptor 1
FoxO1: Forkhead box protein O1
FPR1: Formyl peptide receptor 1
FTH1: Ferritin heavy chain 1
FTL: Ferritin light chain
GPI: Glucose-6pPhosphate isomerase
GPX4: Glutathione peroxidase 4
GSK3β: Glycogen synthase kinase 3 beta
HIF-1α: Hypoxia-inducible factor 1 alpha
HO-1: Heme oxygenase-1
IGF-2: Insulin-like growth factor 2
IGF2BP3: Insulin-like growth factor 2 mRNA-binding protein 3
IL-1β: Interleukin-1β
IL-6: Interleukin-6
iNOS: Inducible nitric oxide synthase
JAK: Janus kinase
JNK: C-Jun NH_2_-terminal kinase
KIF15: Kinesin family member 15
LDHA: Lactate dehydrogenase-A
LKB1: Liver kinase B1
LPS: Lipopolysaccharide
LSD1: Lysine-specific demethylase 1
MAPK: Mitogen-activated protein kinase
miR-195-5p: MicroRNA-195-5p
MITF: Microphthalmia-associated transcription factor
MMP-9: Matrix metalloproteinase-9
MPP: 1-Methyl-4-phenylpyridinium
mTOR: Mechanistic target of rapamycin
MyD88: Myeloid differentiation primary response protein 88
NAFLD: Non-alcoholic fatty liver disease
NaV: Voltage-gated Na^+^
NF-κB: Nuclear factor kappa B
NLRP3: NOD-like receptor protein 3
NO: Nitric oxide
NQO1: NAD(P)H: quinone oxidoreductase 1
Nrf2: Nuclear factor erythroid 2–related factor 2
PADI3: Peptidyl arginine deiminase 3
PDK1: 3-Phosphoinositide-dependent protein kinase-1
PD-L1: Programmed death-ligand 1
PE: Petroleum ether
PGC-1α: Proliferator-activated receptor gamma coactivator 1-alpha
PI3K: Phosphatidylinositol-3-kinase
PIK3CA: Phosphatidylinositol-4, 5-bisphosphate 3-kinase catalytic subunit alpha
PlGF: Placental growth factor
PM2.5: Particulate matter 2.5
p-PKC: Phosphorylated protein kinase C
PS1: Presenilin 1
RANKL: Receptor activator of nuclear factor kappa-B ligand
ROS: Reactive oxygen species
S100A8/A9: S100 calcium binding protein A8/A9
SAR: Structure-activity relationships
SASP: Senescence-associated secretory phenotype
SIRT1: Sirtuin 1
SLC7A11: Solute carrier family 7 member 11
Smad3: Mothers against decapentaplegic homolog 3
SPHK1: Sphingosine kinase 1
Src: Proto-oncogene tyrosine-protein kinase Src
STAT1/3: Signal transducer and activator of transcription 1/3
Tau: Microtubule-associated protein
TCM: Traditional Chinese Medicine
TFEB: Transcription factor EB
TFF2: Trefoil factor 2
TFR1: Transferrin receptor 1
TGF-β: Transforming growth factor-beta
TGR5: Takeda G protein-coupled receptor 5
Th1/2: T helper cell type 1/2
TLR4: Toll-like receptor 4
TNF-α: Tumor necrosis factor-alpha
TPI1: Triosephosphate isomerase 1
TRAF6: TNF receptor-associated factor 6
TRPV1: Transient receptor potential vanilloid 1
VEGF-A: Vascular endothelial growth factor A
VNN1: Vanin-1
VSMC: Vascular smooth muscle cell
YY1: Yin Yang 1
